# Site-Specific DBCO Modification of DEC205 Antibody for Polymer Conjugation

**DOI:** 10.3390/polym10020141

**Published:** 2018-02-02

**Authors:** Simone Beck, Jennifer Schultze, Hans-Joachim Räder, Regina Holm, Meike Schinnerer, Matthias Barz, Kaloian Koynov, Rudolf Zentel

**Affiliations:** 1 Institute of Organic Chemistry, Johannes-Gutenberg University Mainz, Duesbergweg 10-14, D-55128 Mainz, Germany; becksi@uni-mainz.de (S.B.); rholm@uni-mainz.de (R.H.); barz@uni-mainz.de (M.B.); 2 Graduate School Materials Science in Mainz, Staudingerweg 9, D-55128 Mainz, Germany; 3 Max-Planck-Institute for Polymer Research, Ackermannweg 10, D-55128 Mainz, Germany; schultze@mpip-mainz.mpg.de (J.S.); raeder@mpip-mainz.mpg.de (H.-J.R.); koynov@mpip-mainz.mpg.de (K.K.); 4 Institute of Physical Chemistry, Jakob Welder Weg 11, D-55128 Mainz, Germany; mschin@uni-mainz.de

**Keywords:** cancer immune therapy, vaccination, dendritic cells (DCs), pDNA polyplex, RAFT polymerization, targeting, DEC205 antibody, bioorthogonal chemistry, strain-promoted alkyne-azide cycloaddition (SPAAC)

## Abstract

The design of multifunctional polymer-based vectors, forming pDNA vaccines, offers great potential in cancer immune therapy. The transfection of dendritic immune cells (DCs) with tumour antigen-encoding pDNA leads to an activation of the immune system to combat tumour cells. In this work, we investigated the chemical attachment of DEC205 antibodies (aDEC205) as DC-targeting structures to polyplexes of P(Lys)-*b*-P(HPMA). The conjugation of a synthetic block copolymer and a biomacromolecule with various functionalities (aDEC205) requires bioorthogonal techniques to avoid side reactions. Click chemistry and in particular the strain-promoted alkyne-azide cycloaddition (SPAAC) can provide the required bioorthogonality. With regard to a SPAAC of both components, we firstly synthesized two different azide-containing block copolymers, P(Lys)-*b*-P(HPMA)-N_3_(stat) and P(Lys)-*b*-P(HPMA)-N_3_(end), for pDNA complexation. In addition, the site-specific incorporation of ring-strained dibenzocyclooctyne (DBCO) moieties to the DEC205 antibody was achieved by an enzymatic strategy using bacterial transglutaminase (BTG). The chemical accessibility of DBCO molecules within aDEC205 as well as the accessibility of azide-functionalities on the polyplex’ surface were investigated by various SPAAC experiments and characterized by fluorescence correlation spectroscopy (FCS).

## 1. Introduction

Within the field of nanomedicine, cancer therapy and diagnostics are the most active research areas [1,2,3,4,5]. Pathophysiological abnormalities of the tumour tissue, e.g., leaky vasculature and reduced lymphatic drainage, can lead to a passive accumulation of nanoparticles in these regions. This so called EPR (enhanced permeability and retention) effect [6,7,8] can thus increase local drug concentrations at tumour site, while off-target effects can be effectively reduced. Furthermore, nano-sized drug delivery systems enhance the bioavailability of poorly soluble hydrophobic drugs or even highly sensitive therapeutic compounds, such as DNA, RNA or proteins [4,5,9].

Nevertheless, tumour accumulation via EPR effect of nanomedicines highly depends on the nature of the tumour itself and may differ substantially among individual patients [8,10,11]. In comparison to the primary tumour, the treatment of tumour-derived metastasis provides another major challenge for nanomedicines [12,13]. Thus, tumour therapy requires a strategy not only to eliminate cells of solid tumours at a certain position but also to reach migrated tumour cells which are involved in the formation of metastasis, spread within the body. In this respect, the concept of nanocarrier-based cancer immunotherapy seems very attractive [14], by triggering an appropriate immune stimuli to activate the immune system combating tumour cells.

In general, the immune system is involved in combating foreign pathogens in the range of several to hundreds of nanometres and also in eliminating defective endogenous cells. With regard to tumour development, the immune system possesses, however, autoregulatory mechanisms, which often result in immune tolerance, suppressing the immune system to combat tumour cells. Therefore, the concept of anticancer immunotherapy includes the activation of the immune system on the one hand and the reduction of immune tolerance on the other. To address the immune system most effectively, it is thereby necessary to targeted the most sensitive and “powerful” immune cell populations [15,16]. In analogy with nanometre-sized pathogens, like viruses or bacteria, multifunctional nanoparticles may provide a promising platform to interact with immune cells [17].

Especially in the area of activating the immune system for tumour therapy, the application of therapeutic nucleic acids, such as DNA vaccines, represents a promising approach [18,19,20]. This concept involves the transfection of dendritic immune cells (DCs) with tumour antigen encoding plasmid DNA (pDNA), triggering a specific anti-tumour immune response. In general, successfully transfected DCs may express the encoded antigen after transcriptional and translational processes and present several antigen fragments via MHCII complexes to CD4^+^ T cells (T helper cells). Cross-presentation to CD8^+^ DCs leads to antigen presentation via MHCI complexes, resulting in the activation of CD8^+^ T cells which differentiate into cytotoxic T cells (CTLs) and enable a tumour antigen-specific immune response [21]. An advantage of pDNA-vaccines is the long-term persistence of the antigen in the body. After transfection of only a few hundred DCs, the antigen is expressed continuously for several weeks, which is beneficial for inducing profound immune response [22,23]. The fact that only a small number of transfected DCs is sufficient, demonstrates again the advantage of targeting the immune system instead of directly addressing tumour cells in a classical drug-delivery approach. Besides DC-specific promotors [24] within the pDNA construct, which ensure only DC specific antigen expression, the additional incorporation of immune-stimulatory elements, such as CpG-rich motifs [25], leads to optimal DC activation.

For successful in vivo DC-transfection in tumour therapy, a functional nanocarrier system needs to enable effective pDNA delivery. Such polymer-based vectors need a cationic part to complex polyanionic pDNA, resulting in polyion complexes (PICs), called polyplexes. The inclusion of sensitive pDNA in nanodimensional formulations ensures the protection of pDNA from hydrolytic and enzymatic degradation during the delivery through the blood stream. To prevent undesired aggregation due to unspecific interaction of charged polyplexes with serum components, a hydrophilic, non-ionic corona within the nanocarrier is required to ensure effective shielding (stealth-like properties) of the polyplex [26]. Several pDNA carrier systems, based on block copolymers, such as PLys-*b*-PEG [27,28,29], PLys-*b*-PSar [30,31] and PLys-*b*-PHPMA [32,33] have demonstrated their potential to functional pDNA transfer.

To achieve a cell-type specific pDNA transfection, the nanocarrier system can further be equipped with targeting moieties, like receptor ligands or antibodies. A promising DC-targeting approach was shown using mannosylated nanocarriers to target the mannose receptor, expressed on DCs [34,35]. Nevertheless, mannose may possess limited applicability for a systemic application. Especially for the desired immune cell population of CD8^+^ DCs, the specific surface receptor DEC205, a C-type lectin receptor, seems to be a promising target, which can be addressed by a commercially available DEC205 antibody [34,36,37,38].

**Scheme 1:**
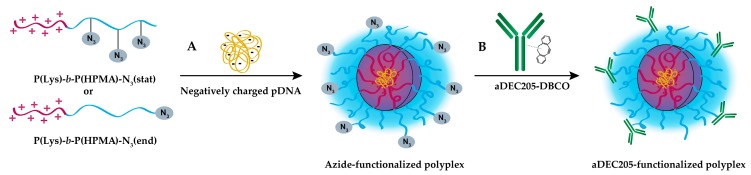
(**A**) Polyplex formation of P(Lys)-*b*-P(HPMA)-N_3_(stat) or P(Lys)-*b*-P(HPMA)-N_3_(end) block copolymers with pDNA. (**B**) Strain-promoted alkyne-azide cycloaddition (SPAAC) of azide-functionalized polyplex and DBCO-modified aDEC205.

In general, the specific conjugation chemistry of such targeting moieties like small molecules (e.g., mannose), bioactive peptides or even antibodies, have a profound effect on the pharmacokinetic profile and the biological activity [39,40,41]. Therefore, in this work, we developed a bioorthogonal conjugation strategy to attach DEC205 antibodies as targeting moieties for DCs to preformed polyplex structures as potential vaccines in tumour immune therapy (Scheme 1). The synthesis of P(Lys)-*b*-P(HPMA) was performed via the reactive ester approach by RAFT (reversible addition-fragmentation chain-transfer) polymerization of pentafluorophenyl methacrylate (PFPMA) using polylysine as macromolecular chain-transfer agent (CTA) [32,42]. This strategy using reactive PFPMA precursor polymers enables great versatility in the synthesis of multifunctional HPMA-based polymers, especially the introduction of bioorthogonal groups via aminolysis in polymer-analogous reactions [43,44].

**Scheme 2:**
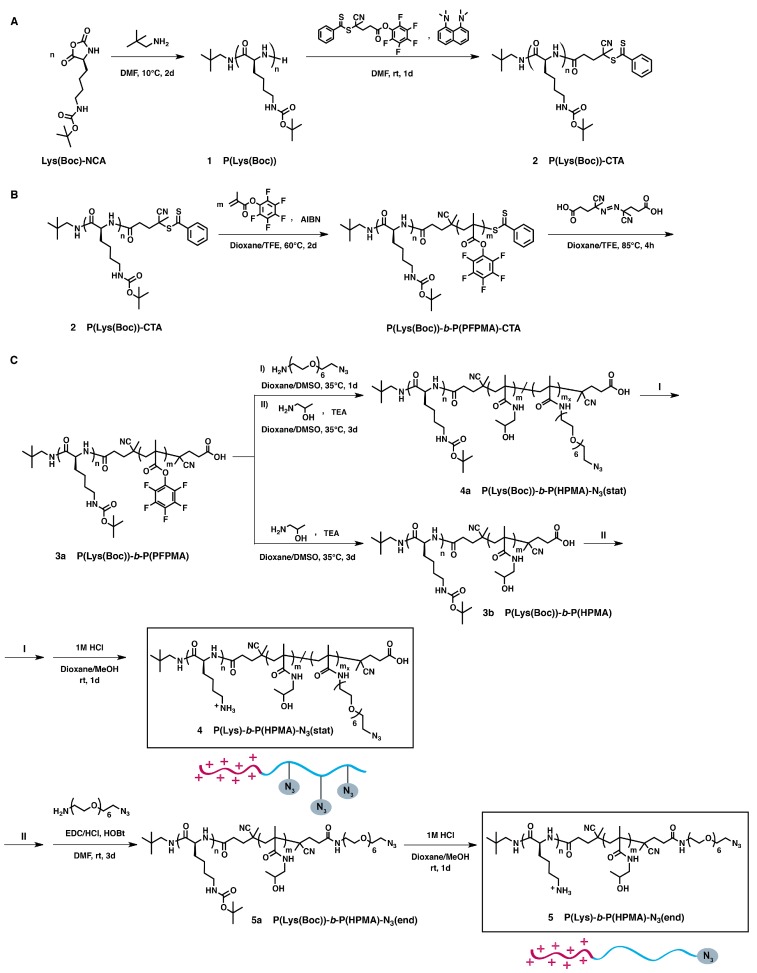
(**A**) Synthesis of P(Lys(Boc)) (1) by ring-opening polymerization of Lys(Boc)-NCA and formation of macromolecular chain transfer agent P(Lys(Boc))-CTA (2). (**B**) RAFT polymerization of PFPMA leading to P(Lys(Boc))-*b*-P(PFPMA) precursor block copolymer (3a). (**C**) Various post-polymerization reactions leading to P(Lys)-*b*-P(HPMA)-N_3_(stat) (4) and P(Lys)-*b*-P(HPMA)-N_3_(end) (5).

For the conjugation of polyplex and antibody we used the bioorthogonal strain-promoted alkyne-azide cycloaddition (SPAAC) [45,46]. Therefore, we attached dibenzocyclooctyne (DBCO) moieties to the DEC205 antibody and introduced azide groups to the polyplex structures by modification of the block copolymers (Scheme 1). Besides the introduction of several azide groups to the P(HPMA) block via aminolysis, an azide end group modification leads to a P(Lys)-*b*-P(HPMA) block copolymer with one single reactive moiety at a certain position (Scheme 2C).

From the perspective of antibody modification, conventional chemical modification strategies for the incorporation of bioorthogonal functionalities, linker molecules or even drug molecules are difficult to control in number and conjugation site [47,48]. This leads to heterogeneous conjugates with different stoichiometries, which may vary in their pharmacokinetic properties [49,50]. To overcome these limitations, we used a site-specific enzymatic modification developed for IgG antibodies, resulting in homogenous conjugates with two functional units at two defined amino acid positions [51]. In a two-step enzymatic synthesis using peptide-*N*-glycosidase F (PNGase F) for a preceding deglycosylation, two lysine-surrogates can be connected to each glutamine residue (Q295) in the heavy chain of the antibody via the enzyme bacterial transglutaminase (BTG).

In this work, we adapted this attractive site-specific strategy to introduce DBCO moieties to the DEC205 antibody for a bioorthogonal SPAAC and report the modification of azide-functionalized P(Lys)-*b*-P(HPMA) polyplexes for a potential application as pDNA vaccines (Scheme 1).

## 2. Experimental Section

### 2.1. Materials

All chemicals and solvents were purchased from common suppliers, such as Sigma-Aldrich (Taufkirchen, Germany) and Acros Organics (Geel, Belgium) and used without further purification unless otherwise indicated. THF and 1,4-dioxane were freshly distilled from Na and DCM from CaH_2_, respectively. DMF was dried over BaO and molecular sieve (3 Å) and fractionally distilled in vacuo. Prior to use, DMF was degassed in vacuo. Diethyl ether was distilled prior to use to remove stabilizer. *N*-ε-Boc-l-lysine was purchased from ORPEGEN (Heidelberg, Germany). Neopentylamine was purchased from TCI Europe (Zwijndrecht, Belgium), dried over NaOH and fractionally distilled. 2-hydroxy-propylamine was fractionally distilled prior to use. Coupling reagent 1-ethyl-3-(3-dimethylaminopropyl)-carbodiimide hydrochloride (EDC·HCl) was purchased from Carbolution Chemicals (St. Ingberg, Germany). Fluorescent dyes Oregon Green 488 cadaverine, Alexa Flour 647 NHS ester, Alexa Fluor 647 DIBO Alkyne and Texas Red cadaverine were purchased from Invitrogen (Carlsbad, CA, USA). 5/6-Carboxyrhodamine 110-PEG_3_-Azide was purchased from Jena Bioscience (Jena, Germany). Monoclonal antibody aDEC205 was purchased from Bio X Cell (West Lebanon, NH, USA). PNGase F was purchased from New England Biolabs (Frankfurt, Germany) and bacterial transglutaminase from ZEDIRA (Darmstadt, Germany). pDNA (pGL3 vector) for polyplex formation was purchased from Promega GmbH (Mannheim, Germany). Coomassie^®^ Brilliantblau R-250 for SDS staining was purchased from neoLab (Heidelberg, Germany). Dialysis was performed using Spectra/Por^®^ 3 membranes (MWCO 3500 or 14,000 g/mol) obtained from Carl Roth (Karlsruhe, Germany). Amicon^®^ Ultra 0.5 mL and 2 mL Centrifugal Filters, MWCO 100K were purchased from Merck (Darmstadt, Germany).

### 2.2. Characterization

400 MHz ^1^H and ^19^F NMR spectra were recorded on a Bruker Avance II 400 and analysed using MestReNova 10.0 software (Mestrelab Research Lab S. L., Santiago de Compostela, Spain, 2015). Gel permeation chromatography (GPC) of polymers was performed in hexafluoroisopropanol (HFIP) containing 3 g/L potassium trifluoroacetate at 40 °C. The flow rate was set to 0.8 mL/min. The columns were packed with modified silica (PFG columns; particle size: 7 µm, porosity: 100 and 1000 Å, respectively. Polymers were detected using a refractive index detector (G1362A RID by JASCO) and a UV/Vis detector (UV-2075 Plus by JASCO). Calibration was conducted using PMMA standards (Polymer Standards Services GmbH (PSS)) with toluene as internal standard. Elution diagrams were analysed using the WinGPC UniChrome 8.00 (Build 994) software (PSS Polymer Standards Service GmbH, Mainz, Germany, 2011). UV/Vis spectra were recorded on a JASCO V-630 UV/Vis spectrophotometer. Antibody samples were measured in PBS pH 7.4. SDS-PAGE analysis was performed using NuPAGE^TM^ 4–12% Bis Tris protein gels, 20× Bolt^TM^ MES SDS Running Buffer, 4× Bolt^®^ LDS Sample Buffer and Novex^®^ Sharp Pre-Stained Protein Standard; all products were purchased from Thermo Fisher Scientific (Waltham, MA, USA). Electrophoresis was conducted in a Mini Gel Tank, XCell Sure Lock^TM^ Mini. Attenuated total reflectance Fourier transformed infrared (ATR-FTIR) spectroscopy was performed on a FT/IR-4100 (JASCO Deutschland GmbH, Pfungstadt, Germany) with an ATR sampling accessory (MIRacleTM, Pike Technologies, Madison, WI, USA). IR spectra were analysed using Spectra Manager 2.0 (JASCO Corporation). Mass spectrometry with electrospray ionization (ESI-MS) was performed on an Aligent 6545 QTOF-MS.

### 2.3. Synthesis of Azide-Functionalized Block Copolymers: P(Lys)-b-P(HPMA)-N_3_(stat) and P(Lys)-b-P(HPMA)-N_3_(end) (SI, Experimental Section)

### 2.4. Synthesis of Dibenzocyclooctyne (DBCO) Linker for DEC205 Antibody Modification (Scheme 4 ***(8)***)

#### 2.4.1. Dibenzocyclooctyne-Pentafluorophenylester: DBCO-PFP Ester (Scheme 4 **(6)**)

In a two-neck round bottom flask, 190 mg (0.57 mmol, 1 eq) of dibenzocyclooctyne-acid were dissolved in 12 mL of abs. THF. Under an argon atmosphere 0.16 mL (1.14 mmol, 2 eq) of triethylamine and 0.20 mL (1.14 mmol, 2 eq) of pentafluorophenyl trifluoroacetate were added slowly via syringe. The reaction mixture was stirred at room temperature for 4 h. After complete conversion as determined by TLC, the solvent was evaporated under reduced pressure. The remaining crude product was dissolved in DCM and extracted twice with millipore water and once with brine. After phase separation, the organic layer was dried with MgSO_4_ and the solvent was removed by rotary evaporation, resulting in a brown oil. For further purification, column chromatography (eluent cyclohexane/ethyl acetate (4:1)) was performed, obtaining 270 mg (0.54 mmol, 95%) of DBCO-PFP ester as a yellow oil.

**^1^H NMR** (400 MHz, CDCl_3_): δ [ppm] = 7.71 (d, 1H, Ar*H*), 7.44–7.23 (m, 7H, Ar*H*), 5.18 (d, 1H, cyclooctinyl-C*H*_2_), 3.69 (d, 1H, cyclooctinyl-C*H*_2_), 2.45 (t, 2H, -C*H_2_*-COO-), 2.30–2.23 (m, 1H, -C*H*_2_-CO-N), 1.98–1.91 (m, 1H, -C*H*_2_-CO-N), 1.57–1.48 (m, 4H, -C*H_2_*-C*H_2_*-).

**^19^F NMR** (400 MHz, CDCl_3_): δ [ppm] = −153.89 (d, 2F, *o*-Ar*F*), −159.39 (t, 1F, *p*-Ar*F*), −163.60 (d, 2F, *m*-Ar*F*).

#### 2.4.2. Dibenzocyclooctyne-*N*-ε-Boc-l-lysine: DBCO-Lys(Boc) (Scheme 4 **(7)**)

250 mg (0.50 mmol, 1 eq) of DBCO-PFP ester were transferred into a *Schlenk* tube and dissolved in 10 mL of dry DMF under an argon atmosphere. 123 mg of *N*-ε-Boc-l-lysine (0.50 mmol, 1 eq) and 174 µL of *N*,*N*-diisopropylamine (DIPEA) (1.00 mmol, 2 eq) were dissolved in abs. DMF and then added dropwise into the DBCO-PFP ester solution. The reaction was completed after 24 h at 40 °C. The solvent was subsequently removed in high vacuum and the crude product was dissolved in ethyl acetate, followed by aqueous extraction once with 5% citric acid and three times with brine. The organic phase was dried with MgSO_4_ and the solvent was removed by rotary evaporation. Finally, the remaining crude product was purified by column chromatography (eluent chloroform/methanol/water (100:10:1)) obtaining 194 mg (0.35 mmol, 69%) of DBCO-Lys(Boc) as a colourless solid.

**^1^H NMR** (400 MHz, CDCl_3_): δ [ppm] = 7.68 (d, 1H, Ar*H*), 7.41–7.16 (m, 7H, Ar*H*), 6.69 (m, 1H, -N*H*-CO-), 5.17 (d, 1H, cyclooctinyl-C*H*_2_), 4.42 (s, 1H, -NH-C*H*-COOH), 3.69 (d, 1H, cyclooctinyl-C*H*_2_), 3.09 (s, 2H, -C*H*_2_-NH-COOC(CH_3_)), 2.31–1.25 (m, 14H, -N-CO-C*H_2_*-C*H_2_*-C*H_2_*-C*H_2_*- and -CH-C*H_2_*-C*H_2_*-C*H_2_*-), 1.43 (s, 9H, br, -C(CH_3_)).

**ESI-MS**: [*m*/*z*] = 600.24 [M + K]^+^, (calc. 600.38); 584.29 [M + Na]^+^, (calc. 584.27), 562.31 [M + H]^+^, (calc. 562.29), C_32_H_39_N_3_O_6_ (561.67 g/mol).

#### 2.4.3. Dibenzocyclooctyne-l-Lysine: DBCO-Lys (Scheme 4 **(8)**)

In a two-neck round bottom flask, 180 mg (0.32 mmol) of DBCO-Lys(Boc) were dissolved in 1 mL of abs. DCM under an argon atmosphere. After the addition of 0.1 mL triisopropylsilane (5 vol %), DBCO-Lys(Boc) was reacted with 1 mL trifluoroacetic acid. The reaction solution was stirred for 30 min at room temperature. The removal of trifluoroacetic acid was carried out by azeotropic distillation with toluene (three times), followed by final distillation with DCM (three times). The remaining crude product was purified by HPLC (eluent water/acetonitrile (95:5 to 0:100; 60 min) to yield 79.9 mg (0.17 mmol, 54%) of DBCO-Lys as a colourless solid.

**^1^H NMR** (400 MHz, DMSO-d_6_): δ [ppm] = 8.88–8.85 (m, 1H, Ar*H*), 8.10 (d, 1H, -N*H*-CO-), 8.01–7.99 (m, 1H, Ar*H*), 7.71–7.52 (m, 4H, Ar*H*), 7.33–7.27 (m, 2H, Ar*H*), 5.35 (s, 2H, cyclooctinyl-C*H_2_*-), 4.20–4.15 (m, 1H, -NH-C*H*-COOH), 3.10 (t, 2H, -C*H_2_*-NH_2_), 2.75 (q, 2H, -C*H_2_*-CH-COOH), 2.21 (t, 2H, -C*H_2_*-CO-NH-), 1.78–1.22 (m, 10H, -N-CO-C*H_2_-*C*H_2_*C*H_2_*- and H_2_N-CH_2_-C*H_2_*-C*H*_2_*-*).

**ESI-MS**: [*m*/*z*] = 462.23 [M + H]^+^, (calc. 462.24); C_27_H_31_N_3_O_4_ (461.55 g/mol).

### 2.5. Enzymatic DBCO Modification of aDEC205

The site-specific enzymatic modification of IgG antibodies by bacterial transglutaminase (BTG) was adapted from the literature and modified [51,52].

#### 2.5.1. Alexa Fluor 647-Labeling of aDEC205: aDEC205_AF647_

2 mg of native aDEC205 (c ≈ 2 g/L, 1xPBS, pH 7.4) were transferred into an *Eppendorf* tube and 83.3 µg (21 µL) of Alexa Fluor 647 NHS ester were added via a stock solution in abs. DMSO (6.67 × 10^−8^ mol, 5 eq, c = 4 g/L). The pH of the reaction mixture was adjusted to slightly basic conditions (pH 8–9) using an aqueous solution of NaHCO_3_ (1M). The reaction was conducted at 37 °C for 2–4 h while gently shaking. The excess of fluorescent dye was removed using centrifugation-dialysis (Amicon^®^ Ultra 2 mL Centrifugal Filters, MWCO 100K) in five washing steps with 1 mL of PBS each. The yield of AF647-labeled aDEC205 was 90–95% as determined by UV/Vis spectroscopy.

#### 2.5.2. Deglycosylation of aDEC205 at Asp297 by Peptide-*N*-Glycosidase F (PNGase F): aDEC205_(AF647)_-dg

2 mg of aDEC205 (c = 1 g/L, 1xPBS, pH 7.4) and 2000 U PNGase F (c = 500,000 U/mL) were reacted in a 2 mL *Eppendorf* tube under constant shaking at 37 °C for 4 h. Enzyme was removed using centrifugation-dialysis (Amicon^®^ Ultra 2 mL Centrifugal Filters, MWCO 100K) in five washing steps with 1 mL of potassium-free PBS (pH 8) each. The same deglycosylation protocol was applied for previously AF647-labeled aDEC205. The yield of aDEC205-dg was 70–80% as determined by UV/Vis spectroscopy.

#### 2.5.3. DBCO Modification of aDEC205 at Glu295 by Bacterial Transglutaminase (BTG): aDEC205_(AF647)_-DBCO

In a typical experiment, 492.3 µg (1.07 µmol, 80 eq) of DBCO-Lys dissolved in abs. DMSO were added to a solution of 2 mg aDEC205-dg (c = 1 mg/mL in potassium-free PBS, pH 8) and 12 U bacterial transglutaminase (BTG) (c = 6 U/mL). If necessary, the reaction mixture was adjusted to pH 8.0–8.3 with a 1M aqueous solution of NaHCO_3_. The reaction mixture within the *Eppendorf* tube was heated to 37 °C and left to react overnight while gently shaking. The excess of linker molecule DBCO-Lys as well as the enzyme BTG were removed from the reaction solution using centrifugation-dialysis (Amicon^®^ Ultra 2 mL Centrifugal Filters, MWCO 100K) in five to ten washing steps using 1 mL of PBS each. The same protocol was applied for previously AF647-labeled aDEC205. The yield of DBCO-modified aDEC205 was 50–60% as determined by UV/Vis spectroscopy.

### 2.6. Strain-Promoted Alkyne-Azide Cycloaddition (SPAAC)

#### 2.6.1. SPAAC of aDEC205-DBCO and 5/6-Carboxyrhodamine 110-PEG_3_-Azide (CR-110-N_3_)

40 µg (c = 1.27 g/L) of aDEC205-DBCO in PBS were transferred into an *Eppendorf* tube and 1.53 µg (2.67 nmol, 10 eq) of CR-110-N_3_ were added via a stock solution in abs. DMSO (c = 1 g/L). The reaction was conducted in six freeze-thaw cycles by freezing the reaction mixture at −18 °C over night and thawing the solution for one hour at room temperature under exclusion of light. The excess of fluorescent dye was removed using centrifugation-dialysis (Amicon^®^ Ultra 0.5 mL Centrifugal Filters, MWCO 100K) in five washing steps with 200 µL of PBS each. The corresponding negative control with native aDEC205 and CR-110-N_3_ was performed under the same reaction conditions.

#### 2.6.2. SPAAC of aDEC205_(AF647)_-DBCO and P(Lys)-*b*-P(HPMA)_(OG488)_-N_3_(stat)

40 µg of aDEC205_AF647_-DBCO in PBS (c = 0.32 g/L, 15 eq) were reacted with 0.5 µg (c = 1 g/L, 1 eq) of P(Lys)-*b*-P(HPMA)-N_3_(stat) in PBS. The reaction was conducted in six freeze-thaw cycles by freezing the reaction mixture at −18 °C over night and thawing the solution for one hour at room temperature under exclusion of light. The analogous reaction with P(Lys)-*b*-P(HPMA)_OG488_-N_3_(stat) and the corresponding negative controls with aDEC205_AF647_/aDEC205 without DBCO modification were performed under the same reaction conditions. Removal of unreacted block copolymer was achieved by centrifugation-dialysis (Amicon^®^ Ultra 0.5 mL Centrifugal Filters, MWCO 100K) in five washing steps using 200 µL of PBS each.

#### 2.6.3. SPAAC of aDEC205_AF647_-DBCO and P(Lys)-*b*-P(HPMA)-N_3_(end)

20 µg of aDEC205_AF647_-DBCO in PBS (c = 0.32 g/L, 1 eq) were reacted with 3.5 µg (c = 1 g/L, 1 eq) of P(Lys)-*b*-P(HPMA)-N_3_(end) in PBS. The reaction and purification protocol was the same as described in Section 2.6.2.

#### 2.6.4. SPAAC of N_3_-Polyplex and Alexa Fluor 647 DIBO Alkyne (AF647-DBCO)

In a first step polyplexes of pDNA (pGL3) and both azide-functionalized cationic block copolymers; P(Lys)-*b*-P(HPMA)-N_3_(stat) and P(Lys)-*b*-P(HPMA)-N_3_(end) were formed, respectively. Therefore, 1 µg of pDNA (pGL3) (c = 0.24 g/L) dissolved in water was transferred in an *Eppendorf* tube and another 25 µL of millipore water were added. In a horizontal position of the *Eppendorf* tube, 10.8 µg (3.83·10^−10^ mol, 1 eq, c = 1 g/L in PBS; N/P = 4) of P(Lys)-*b*-P(HPMA)-N_3_(stat) and P(Lys)-*b*-P(HPMA)-N_3_(end) were pipetted to the *Eppendorf* tubes wall, respectively. Just by vortexing for 10 s, polymer and pDNA were mixed and polyplex formation was performed for 2 h at room temperature. Then, 2.87 µg of AF647-DBCO (1.91 × 10^−9^ mol, 5 eq) in a DMSO stock solution (c = 0.75 g/L) were reacted with both preformed polyplex samples, respectively. Two analogous polyplex/AF647-DBCO samples of each block copolymer were reacted in six freeze-thaw cycles and for 1 d at 37 °C, respectively. Subsequently, the samples were purified by centrifugation-dialysis (Amicon Ultra Centrifugal Filters, 0.5 mL, MWCO 100K) to remove unreacted AF647-DBCO.

#### 2.6.5. SPAAC of N_3_-Polyplex and aDEC205_AF647_-DBCO

Polyplex formation was performed as described in Section 2.6.4 with 1 µg of pDNA (pGL3) (3.18 × 10^−13^ mol, 1 eq) and 10.8 µg (c = 1 g/L in PBS; N/P = 4) of P(Lys)-*b*-P(HPMA)-N_3_(stat) block copolymer. Then, 5 µg of aDEC205_AF647_-DBCO (3.18 × 10^−11^ mol, 100 eq*) were added and the reaction proceeded in seven freeze-thaw cycles.

*Assumption: one pDNA molecule per polyplex [53].

### 2.7. High Resolution Mass Spectrometry: hr-MS

The mass spectrometry experiments were performed on a SYNAPT G2-Si instrument (Waters Corp., Manchester, UK) equipped with an electrospray (ESI) ion source. Basic instrument calibration in the mass range 100–10,000 *m*/*z* was done with clusters of red phosphorus by laser desorption/ionization using a MALDI ion source. The MH^+^ peak of leucine enkephalin (556.2771 *m*/*z*) was used as lock mass in ESI mode. The antibody measurements were carried out at a capillary voltage of 3 kV, a sampling cone voltage of 140 V, a source offset of 100 V and a source temperature of 120 °C. Data processing was done with MassLynx software V4.1 (Waters GmbH, Eschborn, Germany, 2005). The deconvolution data were obtained after smoothing, baseline subtraction and centroiding by manual assignment of peak series at different charge states.

200 µg of each DEC205 antibody sample in PBS (aDEC205 native, aDEC205-dg (Section 2.5.2) and aDEC205-DBCO (Section 2.5.3)) was prepared by centrifugation-dialysis (Amicon Ultra Centrifugal Filters, 0.5 mL, MWCO 100K) using 0.2% formic acid in water. This desalting process was repeated 15 times, each with 250 µL of 0.2% formic acid in water. Each antibody sample was recovered in a total volume of 50 µL. Prior to the measurement, a solution of acetonitrile/water with 0.1% formic acid was added to the antibody samples (1:1 vol.) and mixed by sonication for 5 min. The samples were infused at a flow rate of 5 µL/min by a syringe pump (Legato 180 from Kd Scientific, Holliston, MA, USA).

All chemicals for MS measurements and sample preparation were used in LC/MS quality. Acetonitrile (Ultra CHROMASOLV) was purchased from Fluka (Buchs, Switzerland), water from Panreac (AppliChem GmbH, Darmstadt, Germany) and formic acid from Fisher Scientific (Hampton, New Hampshire, NH, USA).

### 2.8. Fluorescence Correlation Spectroscopy: FCS

FCS measurements were performed using a commercial setup (Zeiss, Germany) that consisted of the module ConfoCor 2 and of an inverted microscope (Axiovert 200), equipped with a Zeiss C-Apochromat 40/1.2 W water immersion objective. For excitation of Alexa Fluor 647-labeled species, a 633 nm He/Ne (helium/neon) laser was used and collected fluorescence was filtered through a LP650 long pass emission filter before reaching an avalanche photodiode detector that enables single-photon counting. In case of excitation of Oregon Green 488 or CR-110-N_3_-labeled samples, the setup was changed using the 488 nm line of an argon laser with a LP505 long pass emission filter. All measurements were performed in eight-well polystyrene-chambered coverglass (Laboratory-Tek, Nalge Nunc International, Penfield, NY, USA) as a sample cell.

For each sample, a series of ten measurements (ten seconds each) was performed. The time-dependent fluctuations of the fluorescence intensity δI(t) caused by the diffusion of the fluorescent species through the confocal observation volume were recorded and analysed by an autocorrelation function:

(1) $$G\left(\tau \right)=1+\frac{\langle \delta I\left(t\right)\;\times \;\delta I\left(t+\tau \right)\rangle }{I{\left(t\right)}^{2}}$$

As it has been shown theoretically for an ensemble of *m* different types of freely diffusing fluorescent species, G(τ) has the analytical form of [54].

(2) $$G\left(\tau \right)=1+\frac{1}{N}\overset{m}{\underset{i=1}{\sum }}\frac{f_{i}}{\left(1+\frac{\tau }{\tau_{D,i}}\right)\times \sqrt{1+\frac{\tau }{S^{2}\times \tau_{D,i}}}}$$

Here, *N* is the average number of diffusing fluorescent species in the observation volume, $\tau_{D,\;i\;}$is the diffusion time of the *i*-th species, $f_{i}$ is the fraction of component *i* and *S* is the so-called structure parameter $S=\frac{z_{0}}{r_{0}}$, where $z_{0}$ and $r_{0}$ represent the axial and radial dimension of the confocal volume, respectively. Furthermore, the diffusion time $\tau_{D,\;i\;}$is related to the respective diffusion coefficient $D_{i}$, through $D_{i}=\frac{r_{0}^{2}}{4\times \tau_{D,\;i}}$. The experimental autocorrelation curve G(τ) was fitted yielding the corresponding diffusion time and subsequently, the diffusion coefficient of the fluorescent species. According to the Stokes-Einstein law, the hydrodynamic radii $R_{h}$ can be calculated assuming spherical particles as $R_{h}=\frac{k_{B}\times T}{6\times \pi \times \eta \times D}\;$, with *T* as absolute temperature, $k_{B}$ as Boltzmann constant and η as viscosity of the solvent.

Furthermore, FCS yielded also the fluorescent brightness (*FB*) of the studied species defined as the ratio between the detected average fluorescent intensity and the mean number of fluorescent species in the observation volume, $FB=\frac{\langle I\left(t\right)\rangle }{N}$.

As the value of $r_{0}$ depends on the characteristics of the specific optical setup, a calibration was performed before each measurement with Alexa Fluor 488 for 488 nm excitation and Alexa Fluor 647 for 633 nm excitation as reference standards with known diffusion coefficients.

All fluorescent dye-labeled samples were dissolved and diluted in PBS (pH 7.4) to a concentration of c ≈ 10^−8^ to 10^−9^ mol/L. Sample purification was performed as described in the corresponding SPAAC experiments (Section 2.6).

## 3. Results and Discussion

### 3.1. Synthesis of Azide-Functionalized P(Lys)-b-P(HPMA) Block Copolymers

The aim of this work was the selective, site-specific functionalization of DEC205 antibodies with dibenzocyclooctyne (DBCO) derivatives to allow bioorthogonal conjugation to pDNA polyplexes via strain-promoted alkyne-azide cycloaddition (SPAAC). This bioconjugation strategy requires the introduction of azide moieties into the cationic block copolymer of P(Lys)-*b*-P(HPMA), used to condense polyanionic pDNA. Therefore, the first step was the synthesis of two different azide-functionalized block copolymers: P(Lys)-*b*-P(HPMA)-N_3_(stat) and P(Lys)-*b*-P(HPMA)-N_3_(end) by combined polymerization techniques of reversible addition-fragmentation chain-transfer (RAFT) polymerization and ring-opening polymerization, as well as several polymer-analogous reactions (Scheme 2).

The first step was the synthesis of poly(*N*-ε-(Boc)-l-lysine) (Scheme 2A (1)) by ring-opening polymerization of *N*-ε-(Boc)-l-lysine *N*-carboxyanhydride (Lys(Boc)-NCA) [55] using neopentylamine as initiator. Previous work of Tappertzhofen et al. [32] showed best transfection efficiencies among various P(Lys)-*b*-P(HPMA) block copolymers in HEK-293T cells with a degree of polymerization of X_n_ = 30 for the polylysine block. Consequently, a chain length of about 30 lysine repeating units was aspired, whereas a degree of polymerization of X_n_ = 32 was confirmed by ^1^H NMR spectroscopy underlining the controlled nature of the ROP polymerization. GPC in HFIP displayed a dispersity of Đ = 1.26 for P(Lys(Boc)).

In order to use the polylysine homopolymer as macromolecular chain transfer agent in the subsequent RAFT polymerization, the amino end group was reacted with pentafluorophenyl-4-phenylthiocarbonylthio-4-cyanovalerate (PFP-CTA) leading to P(Lys(Boc))-CTA (Scheme 2A (2)) [32]. Complete end group functionalization was confirmed by ^1^H NMR spectroscopy, comparing the integral of resulting phenyl signals (7.9–7.5 ppm) to the methylene signal of the lysine side chain at 2.86 ppm.

In a typical experiment, RAFT polymerization of the reactive ester monomer pentafluorophenyl methacrylate (PFPMA) was performed using P(Lys(Boc))-CTA as macromolecular chain transfer agent and azobisisobutyronitrile (AIBN) as initiator (Scheme 2B). Subsequent dithioester end group removal with an excess of 4,4-azobis(4-cyanovaleric acid) (ACVA), to avoid side reactions [56], led to P(Lys(Boc))-*b*-P(PFPMA) (Scheme 2C (3a)) precursor block copolymer with carboxyl end functionality [32]. A conversion of 45% for the PFPMA monomer, as determined by ^19^F NMR spectroscopy, resulted in a calculated degree of polymerization of X_n_ = 162 for the PFPMA block. Thus, a block length ratio of 1:5 for P(Lys(Boc))-*b*-P(PFPMA) (Table 1) has been achieved. Integrity of this precursor block copolymer was ensured by GPC in THF.

The general conversion of P(Lys(Boc))-*b*-P(PFPMA) (Scheme 2C (3a)) into different P(Lys(Boc))-*b*-P(HPMA) block copolymers (Scheme 2C (3b, 4a, 5a)) was performed by various post-polymerization modifications using 2-hydroxy-propylamine (HPA) [43,44]. In case of incorporating the fluorescent dye Oregon Green 488 (OG488) into the block copolymer, 1 mol % of Oregon Green 488 cadaverine with amine functionality was used in the post-polymerization step prior to the conversion with HPA.

The following section describes the synthesis of two different azide-containing P(Lys)-*b*-P(HPMA) block copolymers via various polymer-analogous reactions starting from P(Lys)-*b*-P(PFPMA) precursor block copolymers (Scheme 2C). One the one hand several azide moieties were introduced by post-polymerization modification of the P(PFPMA) block using 15 mol % of the linker molecule *O*-(2-aminoethyl)-*O*′-(2-azidoethyl)-pentaethylene glycol (NH_2_-PEG_6_-N_3_) prior to the conversion with HPA. This led to a statistically azide-modified HPMA block within the resulting P(Lys(Boc))-*b*-P(HPMA)-N_3_(stat) block copolymer (Scheme 2C (4a)).

On the other hand, one single azide group in the block copolymer was introduced via end group modification, using the same azide-containing compound: NH_2_-PEG_6_-N_3_. More precisely, for the synthesis of P(Lys(Boc))-*b*-P(HPMA)-N_3_(end) block copolymers (Scheme 2C (5a)), the conversion of the P(PFPMA) block was only performed by using (OG488) and HPA. Afterwards, the azide functionality was introduced by an amide coupling reaction of the primary amine NH_2_-PEG_6_-N_3_ and the carboxyl end group of P(Lys)-*b*-P(HPMA) block copolymer using 1-ethyl-3-(3-dimethylaminopropyl) carbodiimide hydrochloride (EDC·HCl) and 1-hydroxybenzotriazole (HOBt).

**Figure 1:**
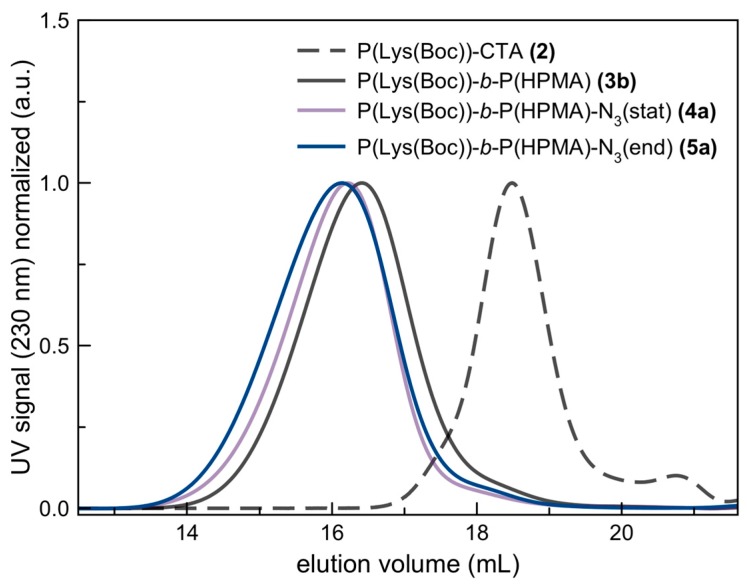
GPC in HFIP of P(Lys(Boc))-CTA (2), P(Lys)-*b*-P(HPMA) (3b), P(Lys)-*b*-P(HPMA)-N_3_(stat) (4a) and P(Lys)-*b*-P(HPMA)-N_3_(end) (5a).

Table 1 displays the characteristics of the different P(Lys(Boc))-*b*-P(HPMA) block copolymers (3b, 4a, 5a), including the macromolecular polylysine chain transfer agent (2). The chain length of 32 repeating units for the P(Lys(Boc))-CTA (2), confirmed by ^1^H NMR spectroscopy, results in a calculated molecular weight of 7687 g/mol. GPC in HFIP (Figure 1, dotted line) shows a narrow distributed homopolymer with a dispersity of 1.26. The slight low-molecular-weight tailing at an elution volume of ~ 21 mL can be explained by the coil to α-helix transition of polylysine with a certain chain length [57].

Characterization of resulting block copolymers by HFIP GPC measurements (Figure 1) displays a shift to smaller elution volumes, which confirms the successful synthesis of P(Lys(Boc))-*b*-P(HPMA) block copolymers with increased molecular weights in the range of 30 000 g/mol (Table 1). Unmodified P(Lys(Boc))-*b*-P(HPMA) (grey line) and both azide-functionalized block copolymers (purple and blue line) show monomodal distributions and display dispersities between 1.4 and 1.6. A block length ratio of 1:5 of P(Lys(Boc)):P(PFPMA) precursor block copolymer was determined by conversion of PFPMA monomer via ^19^F NMR spectroscopy. Molecular weights of resulting P(Lys(Boc))-*b*-P(HPMA) block copolymers were calculated via the appropriate ratio of 1:5 and the corresponding molecular weights of the repeating units. Especially for P(Lys(Boc))-*b*-P(HPMA)-N_3_(stat) and P(Lys(Boc))-*b*-P(HPMA)-N_3_(end) block copolymer, the respective amount of azide-functionalization was considered (Section 3.2).

In order to obtain cationic P(Lys(Boc))-*b*-P(HPMA) block copolymers for polyplex formation, in a last step, the Boc-protective group of the polylysine block of both azide-functionalized block copolymers was removed using HCl-acidic conditions in a methanol/dioxane reaction solution (Scheme 2C, (4,5)). Successful deprotection resulting in P(Lys)-*b*-P(HPMA)-N_3_(stat) and P(Lys)-*b*-P(HPMA)-N_3_(end) block copolymers was confirmed by ^1^H NMR spectroscopy with the disappearance of the characteristic signal of the *tert*-butyl group signal at 1.34 ppm.

### 3.2. Characterization of Azide-Modified Block Copolymers: P(Lys)-b-P(HPMA)-N_3_(stat) and P(Lys)-b-P(HPMA)-N_3_(end)

Azide functionalization of the P(Lys(Boc))-*b*-P(HPMA)-N_3_(stat) block copolymer by post-polymerization modification was confirmed by IR spectroscopy (Figure S1). In comparison to unmodified P(Lys(Boc))-*b*-P(HPMA), the spectrum of P(Lys(Boc))-*b*-P(HPMA)-N_3_(stat) shows an additional signal at a wave number of about 2100 cm^−1^ that is characteristic for the N_3_- bond stretching vibration (ν = 2160–2120 cm^−1^) [58]. Azide end group modification of P(Lys(Boc))-*b*-P(HPMA)-N_3_(end) could not be detected by IR spectroscopy, highly probable due to too small numbers of azide moieties within the large block copolymer.

Therefore, P(Lys(Boc))-*b*-P(HPMA)-N_3_(end) block copolymer was reacted with a dibenzocyclooctyne (DBCO)-modified Texas Red fluorescent dye (SI, 1.5.) in an analytical scale for subsequent GPC characterization. In a previous step, Texas Red cadaverine with amine functionality was modified with dibenzocyclooctyne-PEG_4_-*N*-hydroxysuccinimidyl ester to provide a DBCO moiety within the fluorescent dye for a SPAAC with the azide end group of the block copolymer. The analogous reaction (negative control) was performed with P(Lys(Boc))-*b*-P(HPMA)-N_3_(end) and unmodified Texas Red cadaverine dye to ensure covalent attachment by SPAAC and not due to unspecific ionic or hydrophobic interactions. After removal of unreacted fluorescent dye by dialysis, both samples were analysed by GPC measurements in HFIP by UV detection at 230 nm and 590 nm, respectively (Figure S2).

The GPC elugram of P(Lys(Boc))-*b*-P(HPMA)-N_3_(end) block copolymer shows an absorption maxima at 16.5 mL elution volume for UV detection at 230 nm (Figure S2A, blue solid line), that is based on the amide moieties within the block copolymer. Whereas the same polymer, analysed by UV detection at 590 nm (Texas Red; α_max_ = 596 nm), shows no absorption (Figure S2A, blue dotted line). After the SPAAC reaction of P(Lys(Boc))-*b*-P(HPMA)-N_3_(end) with Texas Red DBCO (positive control), the GPC spectrum now displays a detection at 590 nm (Figure S2B, red solid line) at an elution volume of 16.5 mL, characteristic for the block copolymer. This result confirms a successful SPAAC and therefore, underlines qualitatively the presence of azide end groups in the P(Lys(Boc))-*b*-P(HPMA)-N_3_(end) block copolymer. The negative control experiment of P(Lys(Boc))-*b*-P(HPMA)-N_3_(end) with unmodified Texas Red dye (Figure S2B, grey solid line) shows no significant detection at 590 nm, due to any unspecific dye-polymer interactions.

Furthermore, an additional characterization of P(Lys(Boc))-*b*-P(HPMA)-N_3_(stat) and P(Lys(Boc))-*b*-P(HPMA)-N_3_(end) was performed by ^1^H NMR spectroscopy (Figure S3). In comparison to the spectrum of P(Lys(Boc))-*b*-P(HPMA) without any azide functionality (Figure S3, top), the spectra of P(Lys(Boc))-*b*-P(HPMA)-N_3_(stat) (Figure S3, middle) and P(Lys(Boc))-*b*-P(HPMA)-N_3_(end) (Figure S3, bottom) show an additional signal at 3.51 ppm, respectively. More precisely, the signals between 3.59 ppm and 3.38 ppm correspond to twelve of fourteen methylene groups of PEG units within the NH_2_-PEG_6_-N_3_ linker molecule, used in both azide modification strategies (Scheme 2C).

For P(Lys(Boc))-*b*-P(HPMA)-N_3_(stat) block copolymer, the degree of azide functionalization in the P(HPMA) block was calculated by comparing the integrals of the hydroxyl group at 4.68 ppm with the methylene signals (24 protons) of the azide linker molecule, resulting in 10.5% (Figure S4). With regard to a block ratio of 32 lysine repeating units to 162 HPMA units (1:5), the percentage of 10.5% refers to 17 azide units per block copolymer chain in P(Lys(Boc))-*b*-P(HPMA)-N_3_(stat). Within the given accuracy of the ^1^H NMR spectrum for the methylene signals and the chosen hydroxyl group proton of HPMA, the resulting percentage is in good accordance with the aspired 15 mol % of azide modification (SI, 1.4).

A quantification of azide end group modification of P(Lys(Boc))-*b*-P(HPMA)-N_3_(end) via the corresponding ^1^H NMR spectrum (Figure S5) is rather inaccurate and provides only a very rough estimate, because of the low intensity of the proton signal at 3.51 ppm and partial overlapping with the broad water signal at 3.3 ppm. Nevertheless, a rough calculation leads to a degree of 7% of azide end group modification in P(Lys(Boc))-*b*-P(HPMA)-N_3_(end).

### 3.3. Site-Specific DBCO Modification of aDEC205

**Scheme 3:**
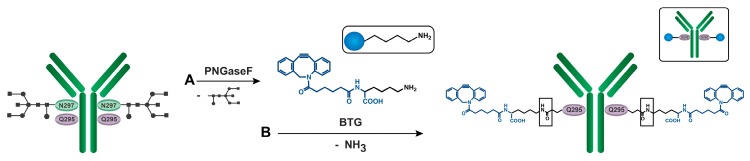
Enzymatic (site-specific) modification of aDEC205. (**A**) Deglycosylation by PNGase F. (**B**) DBCO modification by BTG.

The synthesis of dibenzocyclooctyne (DBCO)-functionalized DEC205 antibodies was performed by an enzymatic, site-specific modification strategy [51]. While conventional protein modification reactions, e.g., using the primary amino group of lysine side chains, lead to heterogeneous conjugates, this enzymatic approach enables the modification of two specific sites (Glu295) within IgG antibodies, resulting in homogenous antibody conjugates (Scheme 3).

We adapted this enzymatic process to introduce DBCO moieties to the DEC205 antibody for further conjugation to azide-containing polyplexes via SPAAC (Scheme 1). The first enzymatic step involves the removal of *N*-linked oligosaccharides at asparagine residue 297 (Asn297/N297) in both heavy chains in the Fc region of the antibody using peptide-*N*-glycosidase F (PNGase F) (Scheme 3A). More precisely, PNGase F belongs to the class of amidases and cleaves between the innermost *N*-acetylglucosmaine (GlcNAc) and asparagine residue of high mannose hybrid carbohydrate structures from *N*-linked glycoproteins, such as in monoclonal antibodies (mAbs) like aDEC205 [59,60,61]. This deglycosylation at N297 leads to an increased mobility of the C/E loop in the Q295-T299 region of mAbs and facilitates the second enzymatic step by bacterial transglutaminase (BTG) (Scheme 3B) [62,63,64].

In general, transglutaminases catalyse the formation of isopeptide bonds between the γ-carboxamide of glutamines and the primary ε-amino group of lysines in peptides by release of ammonia. In this enzymatic antibody modification approach of aDEC205, we used BTG to provide DBCO functionalities in a site-specific manner. The general conditions for the recognition of the appropriate glutamine residue by BTG are very stringent, meaning the glutamine residue must be in a flexible region of the protein and must be surrounded by specific amino acids [65]. Previous work of Schibli and co-workers [51] had shown that BTG can be used for the site-specific modification of both glutamine (Gln295/Q295) side chains in different mAbs. Besides the regioselectivity, that provides homogenous conjugates, another advantage is the modification site at Q295 within the Fc region, which should not affect receptor binding of the antibody within the Fab part.

**Scheme 4:**
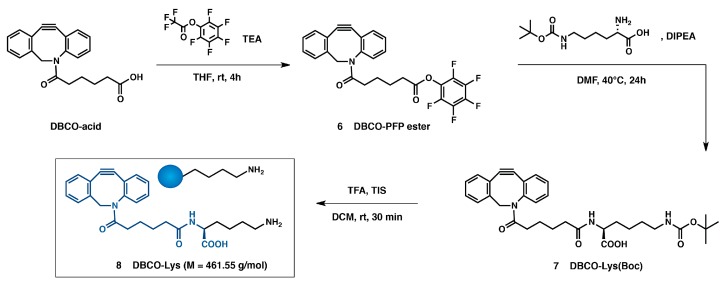
Synthesis of DBCO-Lys.

In contrast to the glutamine recognition, BTG is very flexible for the enzymatic conversion of various lysine surrogates [51]. Therefore, we synthesized a BTG substrate, which contains a DBCO functionality for SPAAC on the one hand and a primary ε-amino lysine group for the recognition of BTG on the other (Scheme 4 (8)).

In a three-step synthesis DBCO-Lys (8) was synthesized starting with DBCO-acid. Activation of the carboxylic acid via the pentafluorophenyl ester approach led to DBCO-PFP-ester (6) and enabled subsequent reaction with the α-amino group of *N*-ε-Boc-l-lysine giving DBCO-Lys(Boc) (7). Finally, the Boc-protective group was removed using acidic reaction conditions in trifluoroacetic acid and dichloromethane. Additional triisopropylsilane (TIS) was used as cation scavenger to trap the intermediary resulting trityl cation of acidic Boc-deprotection to prevent potential side-reactions [66]. In conclusion, DBCO-Lys (8) was successfully synthesized as determined by ^1^H NMR spectroscopy (Figure S6) and finally used in BTG-mediated DEC205 antibody modification.

Characterization of enzymatically deglycosylated and DBCO-functionalized aDEC205 was performed by high resolution mass spectrometry (hr-MS). The DEC205 antibody samples were prepared as described in Section 2.7. For the detection of protein samples, especially for the desired high resolution of high molecular weight proteins (IgGs: ~150 kDa), it is necessary to remove any kind of salt within the sample [67]. Therefore, a thorough desalting procedure was carried out immediately prior to the measurements. Desalting was performed by centrifugation-dialysis using an aqueous solution containing 0.2% of formic acid for at least 15 times by reducing the filter volume from 0.5 mL to 0.1 mL in each step. Besides the solvent exchange from PBS to 0.2% aqueous formic acid, the acidic conditions are mandatory to remove internal salts, which could have been formed by carboxyl—or hydroxyl groups of amino acid side chains with sodium—or potassium ions inside the protein structure. 

Another necessary aspect of using formic acid in the sample preparation, is the protein denaturation, meaning the loss of the tertiary structure, which allows a higher degree of protonation during MS measurements. It should be emphasized that there was no previous protein digestion by proteases, like pepsin [68] or papain [69], leading to antibody fragments. The aDEC205 samples were detected as entire IgG proteins having a molecular weight of approximately 150 kDa. In the electrospray measurements, the signals were detected in the mass range between 2000 and 3500 Da corresponding to a charge state distribution and thus a degree of protonation between 40 and 70. 

**Figure 2:**
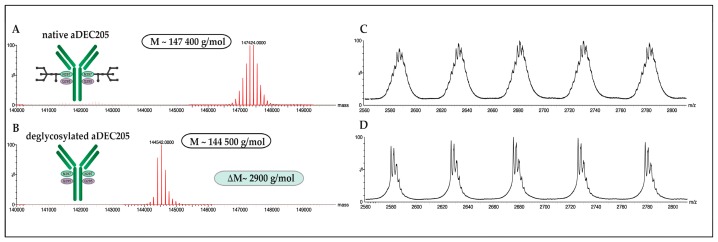
Mass spectra (deconvoluted): (**A**) Native aDEC205. (**B**) Deglycosylated aDEC205. Mass spectra (*m*/*z*) (**C**) Native aDEC205. (**D**) Deglycosylated aDEC205.

The deconvoluted mass spectrum in Figure 2A of native aDEC205 compared to the spectrum of aDEC205 after treatment with PNGase F (Figure 2B) confirms the successful deglycosylation by a shift to lower molecular weights from 147,400 g/mol to 144,500 g/mol. The mass difference of approximately 2900 g/mol corresponds to the range of 2–3 mass% of carbohydrates at Asn297 for IgG antibodies (~150 kDa) [69].

Furthermore, Figure 2C,D shows the *m*/*z*-spectra for both comparative aDEC205 samples and it can be seen nicely that the shape of the signal of deglycosylated aDEC205 (Figure 2D) provides higher resolution, due to a reduced degree of chemical heterogeneity, resulting from the reduced degree of glycosylation in comparison to the signal of the native DEC205 antibody (Figure 2C). This indicates a more uniform protein structure of deglycosylated aDEC205 after removal of post-translational added carbohydrates as compared to the native antibody.

**Figure 3:**
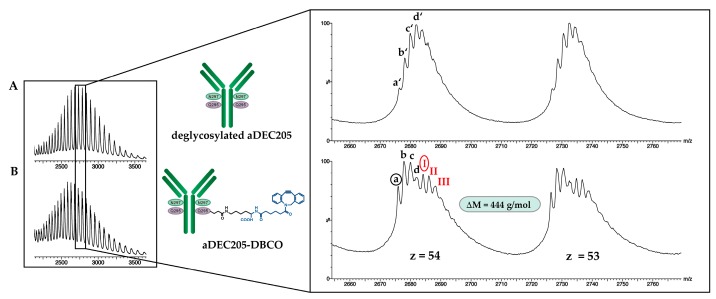
Mass spectra (*m*/*z*): (**A**) Deglycosylated aDEC205. (**B**) DBCO-modified aDEC205.

Besides the analytic proof of successful deglycosylation of aDEC205, the subsequent DBCO modification with DBCO-Lys (Scheme 4) could also be confirmed by hr-MS. Figure 3 shows a section of the *m*/*z*-spectrum of deglycosylated aDEC205 (Figure 3A, top) and the corresponding DBCO-modified aDEC205 sample (Figure 3B, bottom).

Taking a closer look at the signal with a charge state of 54 (z = 54) in the spectrum of DBCO modification (bottom, left), one can identify the first three to four peaks (a–d), matching exactly to the signals (a’–d’) in the spectrum of the deglycosylated antibody sample (top, left).

Furthermore, there is an additional signal sequence (I, II and III) in the signal of DBCO-modified aDEC205 (bottom, left), which was not present in the spectrum of the unmodified, deglycosylated antibody sample. This can be assigned to aDEC205, modified with one DBCO moiety, as determined by the following calculations. Comparing peak (a) with a *m*/*z*-value of 2675.9407 for the sequence of deglycosylated aDEC205 with peak (I): *m*/*z* = 2684.1660 for the additional arising sequence of peaks, a mass difference of 444 g/mol was calculated.

This mass increase of 444 g/mol results from the initial mass of 461 g/mol for DBCO-Lys (Scheme 4), reduced by one molecule ammonia (17 g/mol) per DBCO linkage during isopeptide bond formation with regard to the enzymatic BTG reaction (Scheme 3). Thus, the DBCO modification can be successfully proven but from the signal shape we have to conclude that the modification was achieved only partially and the sample represents mainly a mixture of unmodified (deglycosylated) and singly modified DEC205 antibodies.

Nevertheless, hr-MS of enzymatically modified DEC205 antibodies enables the detection of one single DBCO molecule by a mass difference of only 444 g/mol within a large protein structure of about 147,000 g/mol in highest precision. The spectrum does not provide evidence for antibodies modified by two DBCO molecules. However, the presence of minor amounts cannot be excluded due to the limited chemical resolution.

UV/Vis spectroscopy also provides an alternative method to determine DBCO functionalities at IgG antibodies by specific absorption at 309 nm in addition to the IgG signal at 280 nm [70,71]. Measurements of different DBCO-modified aDEC205 samples in Figure S7A show that the detection of DBCO at 309 nm depends on the amount of DBCO molecules per antibody. The UV/Vis spectrum of enzymatically DBCO-modified aDEC205, where a maximum of one or two DBCO molecules can be attached, did not show any signal at 309 nm. In comparison to the site-specific BTG-mediated modification strategy, aDEC205 was also reacted with DBCO-PEG_4_-NHS in different stoichiometries, to introduce the DBCO moiety non-specifically at various primary ε-amino groups of lysine side chains. By using 20 eq of DBCO-PEG_4_-NHS in a reaction with aDEC205, a signal at 309 nm could be detected, whereas the spectrum with a performance of only 5 eq did not lead to a distinct DBCO signal (Figure S7A).

Besides analytical DBCO detection, UV/Vis spectroscopy was performed to determine antibody concentrations using *Lambert-Beer* law with the specific absorption at 280 nm and the extinction coefficient (ε_(IgG)_ = 203,000 L·mol^−1^·cm^−1^) for IgG antibodies [67]. Furthermore, UV/Vis measurements of AF647-labeled aDEC205 samples determined one fluorescent dye molecule per DEC205 antibody in a quantitative analysis (α_max(AF647)_ = 653 nm; ε_(AF647)_ = 270,000 L·mol^−1^·cm^−1^) (Figure S7B) [72].

Further characterization of enzymatically modified aDEC205 using PNGase F and BTG was performed by SDS PAGE (Figure S8) to ensure complete removal of enzymes and to confirm intact antibody structure without any proteolysis caused by the modification process. The intact DBCO-modified aDEC205 could be detected in one single protein band in a size range of approximately 150 kDa, in analogy to the native DEC205 antibody. The enzymes PNGase F (36 kDa) and BTG (38 kDa) could successfully be removed in five centrifugation-dialysis cycles with 1 mL of PBS each. Therefore, either presence of residual enzyme or even partial degradation of the antibody can be excluded.

The influence of DBCO-modification on the size of the aDEC205, or more precisely on the hydrodynamic radius (R_h_) was characterized by multi-angle dynamic light scattering (DLS) and fluorescence correlation spectroscopy (FCS) for AF647-labeled aDEC205-DBCO (Figures S9 and S10). In terms of measurement accuracy for both experiments, the hydrodynamic radius of aDEC205-DBCO is in the same size range of 5–6 nm compared to the native unmodified DEC205 antibody, meaning there is no significant increase in size after enzymatic modification.

In summary, aDEC205 was successfully functionalized with one DBCO moiety on average via site-specific modification using bacterial transglutaminase (BTG), as determined by high resolution mass spectrometry. Characterization by SDS PAGE confirmed complete removal of enzymes (PNGase F and BTG) and ensured an intact antibody structure without any degradation during the enzymatic modification. Additional fluorescence labeling of aDEC205-DBCO enabled the detection of antibody conjugates by SPAAC experiments in the following performed FCS measurements [73].

### 3.4. Conjugation of Polyplex and aDEC205 via Strain-Promoted Alkyne-Azide Cycloaddition (SPAAC) 

The successful introduction of bioorthogonal groups into both macromolecular structures provided DBCO-functionalized aDEC205 and different azide-modified P(Lys)-*b*-P(HPMA) block copolymers. The next objective was the conjugation of those DEC205-DBCO antibodies with preformed azide-containing pDNA-polyplexes via SPAAC.

#### 3.4.1. SPAAC of aDEC205-DBCO and 5/6-Carboxyrhodamine 110-PEG_3_-Azide (CR-110-N_3_)

**Figure 4:**
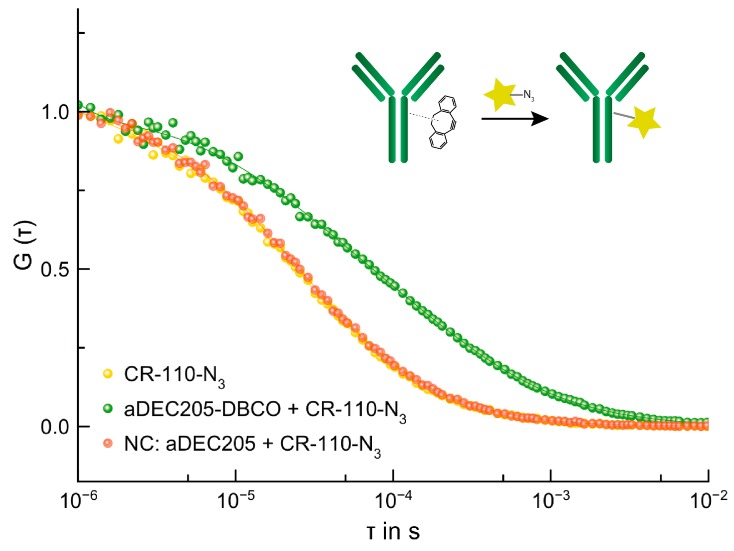
FCS: SPAAC of DBCO-modified aDEC205 and CR-110-N_3-_fluorescence dye: yellow line: CR-110-N_3_: R_h_ = 0.56 nm; green line: SPAAC of aDEC205-DBCO and CR-110-N_3_: R_h_ = 5.8 nm (49%); red line: negative control: native aDEC205 and CR-110-N_3_: R_h_ = 0.58 nm.

First of all, the accessibility of the DBCO group within the DEC205 antibody for SPAAC was analysed by reacting the synthesized aDEC205-DBCO with commercial available (low molar mass) fluorescent dye 5/6-Carboxyrhodamine 110-PEG_3_-Azide. FCS measurements in Figure 4 confirm a successful conjugation of both components via SPPAC by detecting a fluorescent species of R_h_ = 5.8 nm (green line), that is increased in size compared to CR-110-N_3_ fluorescent dye (R_h_ = 0.56 nm; yellow line). Furthermore, the hydrodynamic radius of 5.8 nm for the resulting fluorescent species is in the size range of the DEC205 antibody (Figure S10), which also indicates the attachment of CR-110-N_3_ to aDEC205-DBCO.

The curve characteristics of the green line in Figure 4 for the antibody-dye conjugate differs from the shape of the aDEC_AF647_-DBCO sample in Figure S10, due to the fact that besides the fraction of aDEC205-dye conjugate (5.8 nm-sized species) with 49%, there is almost an equal fraction of excessive free fluorescent dye (51%) within the sample, which was incompletely removed after the reaction.

Additionally, to ensure covalent conjugation of both components via SPAAC and to exclude unspecific ionic or hydrophobic interactions, a negative control (NC) was performed by reacting native aDEC205 without any functional DBCO moiety and CR-110-N_3_ under the same conditions. The red line in Figure 4 shows no fluorescent species with increased size and just overlaps with the yellow line for CR-110-N_3_ (R_h_ = 0.58 nm). This result confirms the covalent attachment of azide-functionalized fluorescent dye to aDEC205-DBCO.

In general, the SPAAC of DBCO-modified aDEC205 with CR-110-N_3_ and subsequent FCS characterization allow a qualitative characterization of BTG-mediated DBCO functionalization of the DEC205 antibody.

Nevertheless, it is important to mention that SPAAC experiments with enzymatically, site-specific DBCO-modified aDEC205 were only successful when the reaction was performed by freeze-thaw cycles as described in Section 2.6. Even the reaction of aDEC205-DBCO and low molecular weight azide fluorescent dye only proceeded in this freeze-thaw procedure and not at room temperature or physiological temperatures (37 °C). This already indicates a very slow conversion of enzymatically DBCO-functionalized aDEC205, probably due to the small amount of only 1–2 DBCO groups per antibody and their limited accessibility and reactivity.

This freeze-thaw method includes the gradual freezing and thawing of an aqueous reaction solution for several times and was already used with sensitive molecules, such as siRNA in polymer conjugation via SPAAC [74]. During the freezing process, the DEC205-DBCO antibody and the corresponding azide component were concentrated by freezing of the aqueous solvent, leading to increased conjugation rates. This unconventional technique enables the conjugation of (bio)macromolecules, like the site-specifically DBCO-modified aDEC205, containing low accessible bioorthogonal groups, due to a small quantity and potential steric hindrance.

#### 3.4.2. SPAAC of aDEC205-DBCO and P(Lys(Boc))-*b*-P(HPMA)-N_3_(stat)

**Figure 5:**
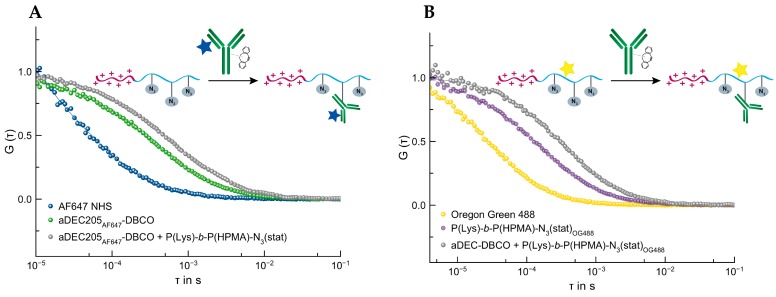
FCS: (**A**) SPAAC of aDEC205_AF647_-DBCO and P(Lys)-*b*-P(HPMA)-N_3_(stat): blue line: AF647 NHS: R_h_ = 0.76 nm; green line: aDEC205_AF647_-DBCO: R_h_ = 6.0 nm; grey line: conjugate of aDEC205_AF647_-DBCO and P(Lys)-*b*-P(HPMA)-N_3_(stat): R_h_ = 10.0 nm (78%). (**B**) SPAAC of aDEC205-DBCO and P(Lys)-*b*-P(HPMA)-N_3_(stat)_OG488_: yellow line: Oregon Green 488: R_h_ = 0.58 nm; purple line: P(Lys)-*b*-P(HPMA)-N_3_(stat)_OG488_: R_h_ = 4.3 nm; grey line: conjugate of aDEC205-DBCO and P(Lys)-*b*-P(HPMA)-N_3_(stat)_OG488_: R_h_ = 10.7 nm (40%).

**Figure 6:**
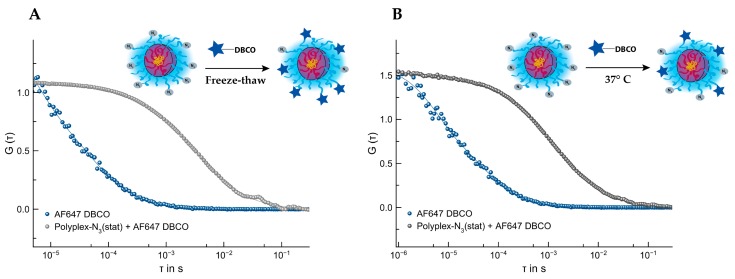
FCS: SPAAC of polyplex by P(Lys)-*b*-P(HPMA)-N_3_(stat) and AF647 DBCO: (**A**) SPAAC via freeze-thaw cycles; blue line: AF647 DBCO: R_h_ = 0.75 nm; light grey line: SPAAC of polyplex: P(Lys)-*b*-P(HPMA)-N_3_(stat) and AF647-DBCO: R_h_ = 52 nm (97%). (**B**) SPAAC at 37 °C; blue line: AF647 DBCO: R_h_ = 0.75 nm; dark grey line: SPAAC of polyplex: P(Lys)-*b*-P(HPMA)-N_3_(stat) and AF647-DBCO: R_h_ = 50 nm (90%).

The following FCS measurements in Figure 5 show the SPAAC of aDEC205-DBCO with the synthesized cationic block copolymer P(Lys)-*b*-P(HPMA)-N_3_(stat) (Scheme 2 (4)), containing several azide moieties in the HPMA block. More precisely, Figure 5A illustrates the successful SPAAC of AF647-labeled aDEC205_AF647_-DBCO with a hydrodynamic radius of 6.0 nm (green line) by an increase to R_h_ = 10.0 nm (grey line) for the corresponding conjugate with the block copolymer P(Lys)-*b*-P(HPMA)-N_3_(stat). The same result was achieved by performing the reaction with P(Lys)-*b*-P(HPMA)-N_3_(stat)_OG488_ and aDEC205-DBCO (Figure 5B), in which the block copolymer is the fluorescence-labeled species. The increase in hydrodynamic radius from 4.3 nm (purple line) for the block copolymer to 10.7 nm (grey line) for the polymer-antibody conjugate is almost similar to the result in Figure 6A. Both experiments were conducted by freeze-thaw cycles.

Additional negative control experiments (not shown) of unmodified aDEC205_AF647_ and aDEC205, respectively with azide-functionalized block copolymer (P(Lys)-*b*-P(HPMA)-N_3_(stat)_OG488_ and P(Lys)-*b*-P(HPMA)-N_3_(stat)_OG488_) proved a conjugation by covalent attachment of polymer and antibody. Furthermore, the same SPAAC reactions were solely successful by the freeze-thaw mechanism and did not lead to any conjugates by conducting the reaction at 37 °C.

In order to establish more homogenous polymer-antibody conjugates, the P(Lys)-*b*-P(HPMA)-N_3_(end) block copolymer (Scheme 2 (5)), containing an azide end group was reacted with aDEC205_AF647_-DBCO under the same reaction conditions than with P(Lys)-*b*-P(HPMA)-N_3_(stat). Nevertheless, FCS measurements of the SPAAC between aDEC_AF647_-DBCO and N_3_-end block copolymer (Figure S11) have not shown any polymer-antibody conjugate within the expected size range of 10 nm, compared to Figure 5A,B. The detected fluorescent species of R_h_ = 7.5 nm simply corresponds in size with the AF647-labeled DEC205 antibody. This failed SPAAC experiment might be explained by the much smaller amount of azide functionalities within the azide end group block copolymer, compared to P(Lys)-*b*-P(HPMA)-N_3_(stat) block copolymer with several azide moieties in the HPMA block.

These results confirm high demands for a successful bioconjugation. Number and accessibility of functional groups within macromolecules like synthetic block copolymer or IgG antibodies, such as aDEC205 are of great importance. Considering their high flexibility in aqueous solution, azide functionalities might be hidden in the coiled polymer conformation, or even DBCO moieties of the DEC205 antibody might be inaccessible due to entrapment into hydrophobic patches. This makes it very challenging to provide the appropriate reaction conditions for a successful SPPAC between two macromolecules.

#### 3.4.3. SPAAC of Polyplex by P(Lys)-*b*-P(HPMA)-N_3_(stat) and AF647 DBCO

In the previous section, we showed the successful conjugation of site-specifically DBCO-modified aDEC205 and P(Lys)-*b*-P(HPMA)-N_3_(stat) block copolymer via SPAAC, performed in freeze-thaw cycles. Prior to the aDEC205-functionalization of polyplexes, consisting of pDNA and P(Lys)-*b*-P(HPMA)-N_3_(stat) block copolymers (Scheme 1), the general accessibility of azide moieties on the polyplex’ surface was analysed.

Therefore, two equal polyplex samples of P(Lys)-*b*-P(HPMA)-N_3_(stat) block copolymer and pDNA (pGL3; 4818 bp) were formed and subsequently reacted with a DBCO containing fluorescent dye (AF647 DBCO) (Section 2.6.4), one at 37 °C and the other by freeze-thaw cycles. For both samples, FCS measurements confirm successful SPAAC of azide-functionalized polyplex and AF647 DBCO (Figure 6A,B).

The fluorescent dye AF647 DBCO (blue line) was detected with a hydrodynamic radius of R_h_ = 0.75 nm and the corresponding conjugates of polyplex and covalently attached AF647-DBCO exhibit a size of R_h_ = 52 nm (A: light grey line) and R_h_ = 50 nm (B: dark grey line), respectively. The size range of R_h_ ≈ 50 nm for polyplexes of P(Lys)-*b*-P(HPMA) with similar block length ratios and pGL3 pDNA is comparable to FCS measurements reported elsewhere [32]. The polyplex samples, based on ionic interactions with negatively charged pDNA, were stable during centrifugation-dialysis for an almost complete removal of unreacted fluorescent dye, resulting in fractions of 97% (A) and 90% (B), respectively for the fluorescence-labeled polyplexes.

These SPAAC experiments in Figure 6 demonstrate a sufficient number and also the general accessibility of azide functionalities on the polyplex’ corona formed with P(Lys)-*b*-P(HPMA)-N_3_(stat) block copolymer for small fluorescent dye molecules. However, the analogous reaction using P(Lys)-*b*-P(HPMA)-N_3_(end) block copolymer in polyplex formation with AF647 DBCO failed (Figure S12), probably due to the same reasons of number and accessibility of azide moieties, discussed in Section 3.4.2.

Especially in the SPPAC of P(Lys)-*b*-P(HPMA)-N_3_(stat)-derived polyplexes and AF647 DBCO, the performance via freeze-thaw cycles, as well as the reaction at 37 °C, led to successful conjugates of polyplex and fluorescent dye (Figure 6). However, the freeze-thaw concept resulted in higher efficiencies with 30 dye molecules per polyplex compared to the reaction at 37 °C with 9 dye molecules per polyplex, respectively. These ratios were determined via FCS using the fluorescence brightness (counts per molecule) of one single AF647 DBCO molecule in relation to the fluorescence brightness of the AF647-labeled polyplex. This result shows a feasible SPAAC at 37 °C of azide-functionalized polyplex and AF647-DBCO on the one hand but an increased conversion proceeding the reaction in freeze-thaw cycles, on the other.

#### 3.4.4. SPAAC of Polyplex by P(Lys)-*b*-P(HPMA)-N_3_(stat) and aDEC205_AF647_-DBCO

The previous performed SPPAC reaction of polyplexes, consisting of cationic block copolymers and sensitive pDNA, with fluorescent dyes (Section 3.4.3 and Figure 6A) by the freeze-thaw process, has not shown any influence on the polyplex structure, like aggregation or polyplex deformation, visualized by FCS measurements. Therefore, the following SPAAC of azide-functionalized polyplexes, derived from P(Lys)-*b*-P(HPMA)-N_3_(stat) block copolymer and AF647-labeled aDEC-DBCO was also performed via the freeze-thaw protocol.

Previous FCS measurements demonstrated, however, that no polyplex-antibody conjugate (R_h_ ≥ 50 nm) was formed if the ratio of polyplex to aDEC205-DBCO was in the range of 1:10 to 1:50.

SPAAC experiments in (Section 3.4.3) confirmed the general accessibility of azide groups on the polyplex’ corona, at least for small DBCO-fluorescent dyes. Consequently, the lack of reactivity between polyplex and antibody is probably mainly effected of the small number of reactive DBCO moieties within aDEC205-DBCO. In addition, the low diffusivity of both components, as a result of their size, is another limiting factor in this SPAAC. Therefore, we increased the amount of DBCO-modified DEC205 antibody (the smaller species; R_h_ ≈ 5–6 nm) in relation to the much bigger polyplex structure (R_h_ ≈ 50 nm) to enhance the probability for the reaction.

**Figure 7:**
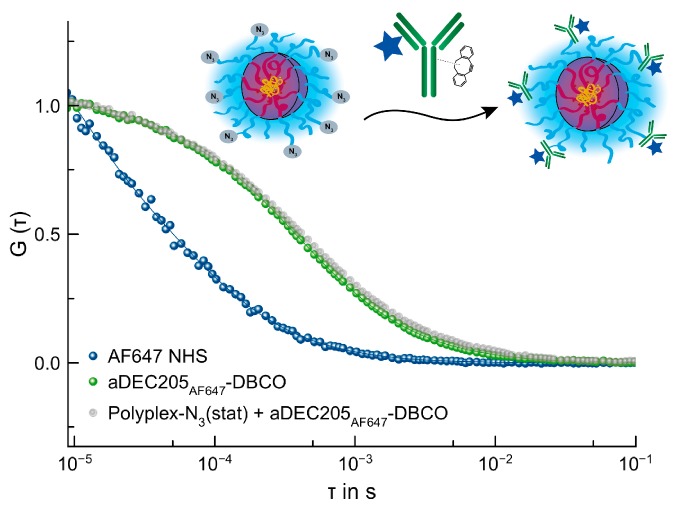
FCS: SPAAC of polyplex by P(Lys)-*b*-P(HPMA)-N_3_(stat) and aDEC205_AF647_-DBCO: blue line: AF647 NHS: R_h_ = 0.76 nm; green line: aDEC205_AF647_-DBCO: R_h_ = 5.5 nm; conjugate of aDEC205-DBCO and polyplex by P(Lys)-*b*-P(HPMA)-N_3_(stat): R_h_ = 52 nm (5%).

Now, using an excess of 100 equivalents of AF647-labeled aDEC205-DBCO in the SPAAC with an azide-functionalized polyplex, a fluorescent species in the polyplex size range of R_h_ = 52 nm (Figure 7, grey line) could be detected via FCS confirming a successful SPAAC, at least for a certain extent. The FCS correlation curves of aDEC205_AF647_-DBCO (green line) and of the polyplex-antibody conjugate (grey line) show an almost identical shape, although they originate from solutions containing species with different hydrodynamic radii: R_h_ = 5.5 nm for aDEC205_AF647_-DBCO compared to R_h_ = 52 nm for the polyplex-antibody conjugate. This is a result of the low fraction of only 5% for the polyplex-antibody species in the reaction mixture and is directly caused by the high excess of unremoved DBCO-modified aDEC205, which was necessary to force the conversion between both macromolecules in this SPAAC.

The successful conjugation of pDNA polyplexes derived from P(Lys)-*b*-P(HPMA)-N_3_(stat) block copolymers and site-specifically DBCO-modified aDEC205, demonstrated here by FCS, is a proof of concept for a feasible bioorthogonal SPAAC between these two macromolecular components. But nevertheless, the high amount of elaborately synthesized DEC205-DBCO antibody (100-fold excess), which is necessary for this reaction and the subsequent problem of removal of unreacted antibody by an appropriate process, limits a broad applicability of this conjugation strategy.

## 4. Conclusions

In this work, aDEC205, which is specific for targeting dendritic immune cells, was site-specifically modified with strained dibenzocyclooctyne (DBCO) groups by an enzymatic strategy [51,52]. These DBCO moieties, introduced by the transglutaminase-based reaction at defined positions in the heavy chains of the DEC205 antibody (glutamine 295), were available for diverse SPAAC reactions.

Furthermore, recently developed cationic block copolymers of P(Lys)-*b*-P(HPMA) are suitable for polyplex formation with pDNA [32]. The synthesis of the P(HPMA) block via the reactive ester approach by RAFT polymerization of PFPMA [42] enabled the facile introduction of azide moieties leading to P(Lys)-*b*-P(HPMA)-N_3_(stat). This azide-functionalized block copolymer was reacted with site-specifically modified aDEC205-DBCO in a bioorthogonal SPAAC resulting in polymer-antibody conjugates. Moreover, we were able to show the accessibility of azide functionalities after polyplex formation of P(Lys)-*b*-P(HPMA)-N_3_(stat) with pDNA in a SPPAC using a small DBCO-modified fluorescent dye.

However, analogous SPAAC experiments with the azide end group modified block copolymer P(Lys)-*b*-P(HPMA)-N_3_(end) failed, most probably, due to a too small number of reactive azide groups within the polymer and limited accessibility.

In general, fluorescence correlation spectroscopy (FCS) was proven to be the ideal method to characterize SPAAC reactions of (bio)macromolecules with small amounts of reactive groups by detecting the increase in size of the respective fluorescent species resulting from the performed conjugation reaction.

In a proof-of-concept experiment, conjugation of azide-functionalized polyplexes (derived from P(Lys)-*b*-P(HPMA)-N_3_(stat) block copolymer) with aDEC205-DBCO via SPAAC could be achieved. Nevertheless, a large excess of aDEC205-DBCO was required to achieve a conversion between both macromolecular components. Besides their low diffusivity due to their sizes (R_h_ ≈ 6 nm for the antibody and R_h_ ≈ 50 nm for the polyplex, respectively), the small amount of reactive DBCO moieties (maximal 1–2 DBCO per DEC205 antibody) contributed to the limited conversion.

Considering—in addition—the large synthetic effort to prepare the site-specifically DBCO-modified DEC205 antibody, the conjugation of block copolymers or even polyplex structures might thus be limited.

In this context, it should be mentioned that our work underlines the concept of site-specific, transglutaminase-mediated antibody modification for the synthesis of homogenous antibody-drug conjugates (ADC) or radioimmune conjugates [51,52] but it cannot be seen as a general antibody modification strategy for further conjugation to nanoparticles by bioorthogonal chemistry.

## Figures and Tables

**Table 1:** Polymer characterization.

Polymer	X_n_	Block Ratio P(Lys):P(HPMA)	Đ (HFIP GPC)	M_n_ (^1^H/^19^F NMR) [g/mol]
P(Lys(Boc))-CTA (2)	32	-	1.26	7687
P(Lys(Boc))-*b*-P(HPMA) (3b)	32:162	1:5	1.41	30,857 *
P(Lys(Boc))-*b*-P(HPMA)-N_3_(stat) (4a)	32:162	1:5	1.53	35,537 */**
P(Lys(Boc))-*b*-P(HPMA)-N_3_(end) (5a)	32:162	1:5	1.58	30,880 */**

* M_n_ was calculated via block length ratio (1:5), determined by ^19^F NMR spectroscopy for P(Lys(Boc))-*b*-P(PFPMA) precursor block copolymer. ** 10% N_3_(stat) and 7% N_3_(end) functionalization included.

## Supplementary Materials

The supporting information (SI) is available online at http://www.mdpi.com/2073-4360/10/2/141/s1.
