# Supplementary material for: Site-Specific DBCO Modification of DEC205 Antibody for Polymer Conjugation
Source: Polymers (Basel). 2018 Feb 2;10(2):141. doi: 10.3390/polym10020141 (PMC6414842; doi:10.3390/polym10020141)
Supplement: Supplementary file 1 [file polymers-10-00141-s001.pdf]

Supplementary Materials: Site-Specific DBCO Modification of DEC205 Antibody for Polymer Conjugation

Simone Beck [1,2], Jennifer Schultze [3], Hans-Joachim Räder [3], Regina Holm [1], Meike Schinnerer [4], Matthias Barz [1], Kaloian Koynov [3] and Rudolf Zentel [1,*]

Experimental Section

1. Synthesis of Azide-Functionalized Block Copolymers: P(Lys)-b-P(HPMA)-N_3(stat) and P(Lys)-b-P(HPMA)-N_3(end)

*1.1. Poly(N-ε-tert-Butyloxycarbonyl-L-Lysine): P(Lys(Boc)) (Scheme 2A **(1)**)*

The synthesis of poly(*N*-ε-*tert*-butyloxycarbonyl-L-lysine) was adapted from the literature [1] and slightly modified. The corresponding monomer *N*-ε-*tert*-butyloxycarbonyl(Boc)-L-lysine-*N*-carboxyanhydride (Lys(Boc)-NCA) was synthesized according to the literature [1].

Under nitrogen counter flow 1.1347 g of Lys(Boc)-NCA (4.17 mmol) were transferred into a *Schlenk* tube and dried in high vacuum for 30 min. To dissolve the NCA monomer, 10 mL of abs. DMF were added via syringe under nitrogen counter flow. Into a separate glass vial, flushed with nitrogen, a stock solution of neopentylamine in DMF was prepared (0.139 mol/L). 1 mL of this solution (16.3 µL neopentylamine; 0.139 mmol) was directly added via syringe into the monomer solution under nitrogen counter flow. The reaction was stirred at 10 °C and was opened to the *Schlenk* line to prevent impurities to enter the reaction vessel while allowing resulting CO_2 to escape. After 2 d, conversion was complete as monitored by IR spectroscopy (disappearance of the characteristic NCA carbonyl peaks at 1853 cm^{-1} and 1786 cm^{-1}). The polymer solution was precipitated in cold distilled diethyl ether and centrifuged. After discarding the supernatant, the precipitated colorless solid was resuspended in distilled diethyl ether by sonication two times and centrifuged again. The polymer was carefully evaporated to dryness by constant air flow and afterwards dissolved in MeOH, suspended in millipore water and lyophilized to obtain 717 mg of P(Lys(Boc)) as colorless powder (3.13 mmol, 75%).

^{1}H NMR (400 MHz, DMSO-d_6): δ[ppm]=8.23-7.90 (br, (1n*H*), -N*H*-CH-CO-), 6.67-6.33 (br, (1n*H*), -N*H*-CO-O-C(CH_3)$_3$), 4.21-3.80 (br, (1n*H*), -CO-C*H*-NH-), 2.88 (br, (2n*H*), -C*H*$_2$-NH-CO-O-C(CH_3)$_3$), 1.78-1.36 (br, (15n*H*) –CH-C*H*$_2$-C*H*$_2$-C*H*$_2$-CH$_2$-NH- and -NH-CO-O-C(C*H*$_3$)$_3$), 0.84-0.82 (9H, -CH$_2$-C(C*H*$_3$)$_3$).

*1.2. Poly(N-ε-tert-Butyloxycarbonyl-L-Lysine)-Chain Transfer Agent: P(Lys(Boc))-CTA (Scheme 2A **(2)**)*

P(Lys(Boc))-CTA was synthesized as previously reported [2]. 513 mg ($6.91\cdot10^{-5}$ mol, 1 eq) of P(Lys(Boc)) were dissolved in 5 mL of dry *N*-methyl-2-pyrrolidon (NMP) under an argon atmosphere. In a separate flask 61.5 mg (1.38×10^{-4} mol, 2 eq) of pentafluorophenyl-4-phenylthiocarbonylthio-4-cyanovalerate (PFP-CTA), synthesized as reported elsewhere [3], and 58.7 mg (2.76×10^{-4} mol, 4 eq) of *N*,*N*,*N'*,*N'*-tetramethyl-1,8-naphthalenediamine were dissolved in 1.5 mL of NMP and added via syringe to the polylysine solution under argon counter flow. The reaction was stirred over night at room temperature under exclusion of light. NMP was removed in high vacuum and the crude product was dissolved in 1 mL of 2,2,2-trifluoroethanol (TFE) and subsequently precipitated in cold distilled diethyl ether. The bright red colored supernatant, containing the excess of unreacted PFP-CTA, was discarded. The equally bright red colored precipitate was dissolved in 3 ml of TFE and purified by column chromatography (eluent ethyl acetate/methanol (10:1; 5:1; 1:1)). After evaporation of solvents, the crude product was dissolved in 2 mL of TFE and precipitated in

millipore water. After lyophilization 513 mg (6.74×10^{-5} mol, 97%) of P(Lys(Boc))-CTA were obtained as a bright red solid.

^{1}H NMR (400 MHz, DMSO-d$_6$): δ[ppm]=8.20 (br, (1n*H*), -N*H*-CH-CO-), 7.92-7.90 (d, 2H, *o*-Ar*H*), 7.72-7.68 (m, 1H, *p*-Ar*H*), 7.54-7.49 (m, 2H, *m*-Ar*H*), 6.66-6.33 (br, (1n*H*), -N*H*-CO-O-C(CH$_3$)$_3$), 4.16-3.78 (br, (1n*H*), -CO-C*H*-NH-), 2.86 (br, (2n*H*), -C*H*$_2$-NH-CO-O-C(CH$_3$)$_3$), 1.86-1.19 (br, (15n*H*) –CH-C*H*$_2$-C*H*$_2$-C*H*$_2$-CH$_2$-NH- and -NH-CO-O-C(C*H*$_3$)$_3$), 0.83-0.81 (9H, -CH$_2$-C(C*H*$_3$)$_3$).

1.3. P(Lys(Boc))-b-P(PFPMA) (Scheme 2C ***(3a)****)*

The synthesis of P(Lys(Boc))-*b*-P(PFPMA) precursor block copolymer, obtained by RAFT polymerization, including dithioester end group removal with 4,4′-azobis(4-cyanovaleric acid) (ACVA), was adapted from the literature [2] and slightly modified. A *Schlenk* tube was loaded with 42.3 mg (5.51×10^{-6} mol, 1 eq) of P(Lys(Boc))-CTA, which was dissolved in a mixture of 0.8 mL TFE and 4 mL of abs. dioxane. 500 mg (1.98×10^{-3} mol, 360 eq) of pentafluorophenyl methacrylate (PFPMA monomer) [4] and 0.301 mg (1.83×10^{-6} mol, 1/3 eq) of the initiator azobisisobutyronitrile (AIBN) were added via stock solution in abs. dioxane under nitrogen counter flow. After three subsequent freeze-pump-thaw cycles, the reaction was performed at 60 °C for 2 d. Conversion of 45% was confirmed by ^{19}F NMR spectroscopy. The polymer solution was precipitated in n-hexane for three times, redissolving the precipitate in a mixture of TFE/dioxane (1:8). The polymer was dried in *vacuo* at 40 °C over night, obtaining 184 mg (3.79×10^{-6} mol) of P(Lys(Boc))-*b*-P(PFPMA) with dithioester end group as a bright red solid. Removal of dithioester end group was performed by reacting 150 mg (3.09×10^{-6} mol) of P(Lys(Boc))-*b*-P(PFPMA), dissolved in TFE/dioxane (1:8) with an excess of 22 mg ($7.73\cdot10^{-5}$ mol, 25 eq) of ACVA at 85 °C for 4 h. The polymer solution was precipitated three times in n-hexane/diethyl ether (2:1) and dried in *vacuo* at 40 °C to obtain 120 mg (2.48×10^{-6} mol, 80%) of P(Lys(Boc))-*b*-P(PFPMA) as a colorless solid.

^{1}H NMR (400 MHz, CDCl$_3$): δ[ppm]=8.21 (br, (1n*H*), -N*H*-CH-CO-), 5.15 (br, (1n*H*), -N*H*-CO-O-C(CH$_3$)$_3$), 3.84 (br, (1n*H*), -CO-C*H*-NH-), 3.08 (br, (2n*H*), -C*H*$_2$-NH-CO-O-C(CH$_3$)$_3$), 2.40-1.91 (br, (2m*H*), -C*H*$_2$-C-CH$_3$), 1.61-1.27 (br, (15n*H*) –CH-C*H*$_2$-C*H*$_2$-C*H*$_2$-CH$_2$-NH- and -NH-CO-O-C(C*H*$_3$)$_3$, (3m*H*) -CH$_2$-C-C*H*$_3$), 0.88 (9H, -CH$_2$-C(C*H*$_3$)$_3$).

1.4. P(Lys(Boc))-b-P(HPMA)-N$_3$(stat) and P(Lys(Boc))-b-P(HPMA)-N$_3$(end) (OG488) (Scheme 2C ***(4a, 5a)****)*

The respective azide-functionalized block copolymers P(Lys(Boc))-*b*-P(HPMA)-N$_3$(stat) and P(Lys(Boc))-*b*-P(HPMA) were synthesized with and without the fluorescent dye OG488.

P(Lys(Boc))-*b*-P(HPMA)-N$_3$(stat): **(4a)**

50 mg (1.03×10^{-6} mol, 1 eq) of P(Lys(Boc))-*b*-P(PFPMA) block copolymer were dissolved in a mixture of 2 mL abs. dioxane and 0.5 mL dry DMSO in a nitrogen-flushed *Schlenk* tube. 60.1 µL of triethylamine (4.34×10^{-4} mol, 421 eq) were added to the polymer solution under nitrogen counter flow. For fluorescence labeling, 0.83 mg of OG488 cadaverine (1.67×10^{-6} mol, 1 mol% of PFPMA block) were transferred into the reaction vessel via a DMSO stock solution (2.5 g/L) and stirred at 35 °C for 4–5 h. Subsequently, 8.78 mg (2.51×10^{-6} mol, 15 mol% of PFPMA block) of NH$_2$-PEG$_6$-N$_3$ were added via a stock solution in DMSO and the reaction was performed at 35 °C over night. The final conversion to Poly(HPMA) was achieved by adding 26 µL (3.34×10^{-4} mol; 324 eq) of 2-hydroxy-propylamine (HPA) and the reaction was stirred for 3 d at 35 °C. To ensure complete conversion of the PFPMA reactive ester block, the reaction was treated with an additional amount of 26 µL HPA (3.34×10^{-4} mol; 324 eq) after the first day. After full conversion was confirmed by ^{19}F NMR spectroscopy, the polymer solution was precipitated once in cold distilled diethyl ether. The crude product was dissolved in DMSO and dialyzed against millipore water (MWCO 14000 g/mol) and lyophilized to obtain 21.5 mg (77%) of P(Lys(Boc))-*b*-P(HPMA)-N$_3$(stat) as colorless (orange colored with OG488) solid.

P(Lys(Boc))-*b*-P(HPMA)-N$_3$(end): **(5a)**

The synthesis of azide end group-functionalized block copolymer was performed in the same way as described for P(Lys(Boc))-*b*-P(HPMA)-N_3(stat), except using NH_2-PEG_6-N_3 in the post-polymerization reaction. 30 mg (0.98 µmol, 1 eq) of the preliminary obtained block copolymer P(Lys(Boc))-*b*-P(HPMA) with carboxyl end group were dissolved in 4 mL of dry DMF under a nitrogen atmosphere and the solution was cooled to 0 °C. Then, 2.7 µL (19.6 µmol, 20 eq) of triethylamine and 3.77 mg (19.6 µmol, 20 eq) of EDC·HCl were added via DMF stock solution under nitrogen counter flow. After 30 min stirring at 0 °C, 2.65 mg (19.6 µmol, 20 eq) of HOBt dissolved in DMF were transferred into the reaction mixture. Finally, after additional 30 min at 0 °C, the polymer was reacted with 6.88 mg (19.6 µmol, 20 eq) of the amine compound NH_2-PEG_6-N_3 for 3 d at room temperature. For further purification, the reaction solution was dialyzed against millipore water containing 5% of citric acid (MWCO 3500 g/mol), saturated sodium hydrogen carbonate, and finally against pure millipore water. After lyophilization 21.6 mg (72%) of P(Lys(Boc))-*b*-P(HPMA)-N_3(end) were obtained as colorless (orange colored with OG488) solid.

^{1}**H NMR** (400 MHz, DMSO-d_6): δ[ppm]=8.35-8.01 (br, (1n*H*), -N*H*-CH-CO-), 7.33-7.19 (br, (1m*H*), -CO-N*H*-CH_2-), 6.65-6.31 (br, (1n*H*), -N*H*-CO-O-C(CH_3)$_3$), 4.68 (br, (1m*H*), (-CH-O*H*), 3.66 (br, (1m*H*), CH_3-C*H*-CH_2-), 3.59 (br, (2m$_x$*H*), -NH-CH_2-C*H*$_2$-O-), 3.51 (br, (20m$_x$*H*), -NH-CH_2-CH_2-O-(C*H*$_2$- C*H*$_2$-O)$_5$-CH_2-CH_2-N_3), 3.38 (br, (2m$_x$*H*), N_3-CH_2-C*H*$_2$-O-), 2.87 (br, (2n*H*/2m*H*), -C*H*$_2$-NH-CO-O-C(CH_3)$_3$/-CO-NH-C*H*$_2$-), 1.34 ((9H), -NH-CO-O-C(C*H*$_3$)$_3$), 1.00 (br, (3m*H*), C*H*$_3$-C-CO-NH-), 2.14-0.67 (br, (6n*H*), –CH-C*H*$_2$-C*H*$_2$-C*H*$_2$-CH_2-NH-) and (2m*H*), CH_3-C-C*H*$_2$-).

1.5. Qualitative Detection of Azide End Group Modification

Positive control: 31.8 µg (7.20 × 10^{-8} mol, 1 eq) of dibenzocyclooctyne-PEG_4-*N*-hydroxysuccinimidyl ester, in a stock solution in dry DMSO (c=10 g/L), were reacted with 49.9 µg (7.20 × 10^{-8} mol, 1 eq) of Texas Red cadaverine in dry DMSO (c=2.5 g/L) over night at room temperature under exclusion of light. 2.2 mg P(Lys(Boc))-*b*-P(HPMA)-N_3(end), dissolved in dry DMSO, were added and the reaction was performed for 3 d while gently shaking under exclusion of light.

Negative control: 49.9 µg (7.20 × 10^{-8} mol, 1 eq) of unmodified Texas Red cadaverine in dry DMSO (c=2.5 g/L) were reacted with 2.2 mg of P(Lys(Boc))-*b*-P(HPMA)-N_3(end) under the same reaction conditions compared to the positive control. Both samples were purified by dialysis against millipore water/methanol (1:1) (MWCO 3500 g/mol).

*1.6. Boc-Deprotection of P(Lys(Boc))-b-P(HPMA)-N_3(stat) and P(Lys(Boc))-b-P(HPMA)-N_3(end) (Scheme 2C **(4, 5)**)*

The removal of the Boc-protective group of the polylysine block in both azide-functionalized block copolymers, P(Lys(Boc))-*b*-P(HPMA)-N_3(stat) and P(Lys(Boc))-*b*-P(HPMA)-N_3(end), was adapted from the literature [2] and slightly modified. 16 mg of P(Lys(Boc))-*b*-P(HPMA)-N_3(stat) and P(Lys(Boc))-*b*-P(HPMA)-N_3(end), respectively were stirred at room temperature over night in 1M HCl in 2 mL of dioxane/MeOH. The solvents were evaporated under reduced pressure and hydrochloric acid was removed by azeotropic distillation with toluene (three times) and additional three times with DCM. The crude product was dissolved in DMSO, dialyzed against millipore water (MWCO 3500 g/mol) and lyophilized to obtain 10.5 mg (66%) of deprotected P(Lys)-*b*-P(HPMA)-N_3(stat) and P(Lys)-*b*-P(HPMA)-N_3(end), respectively.

^{1}**H NMR** (400 MHz, DMSO-d_6): δ[ppm]=8.33-8.00 (br, (1n*H*), -N*H*-CH-CO-), 7.65-7.10 (br, (1m*H*), -CO-N*H*-CH_2-), 4.70 (br, (1m*H*), (-CH-O*H*), 3.67-3.37 (br, (1m*H*), CH_3-C*H*-CH_2- and (14m$_x$*H*), -NH-CH_2-C*H*$_2$-O- and -NH-CH_2-CH_2-O-(C*H*$_2$-C*H*$_2$-O)$_5$-CH_2-CH_2-N_3 and N_3-CH_2-C*H*$_2$-O-), 2.92 (br, (2n*H*/2m*H*), -C*H*$_2$-NH-CO-O-C(CH_3)$_3$/-CO-NH-C*H*$_2$-), 2.01-0.84 (br, (3m*H*), C*H*$_3$-C-CO-NH- and (6n*H*) –CH-C*H*$_2$-C*H*$_2$-C*H*$_2$-CH_2-NH-) and (2m*H*) CH_3-C-C*H*$_2$-).

Results

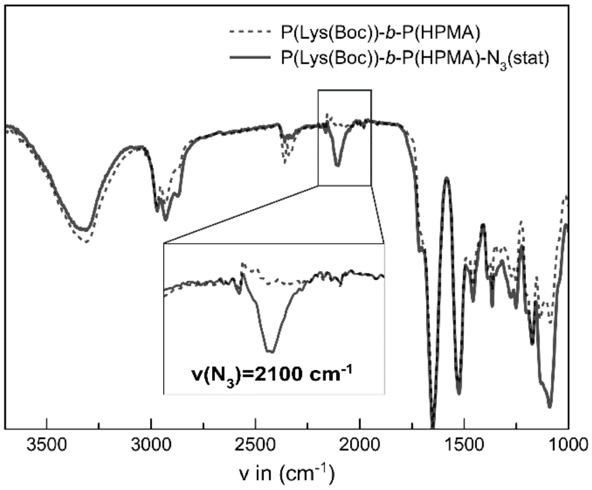

Figure S1. IR spectrum of P(Lys(Boc))-*b*-P(HPMA) (dotted line) and P(Lys(Boc))-*b*-P(HPMA)-N_3(stat) (solid line) with characteristic signal at ~2100 cm^{-1} for the N_3-bond stretching vibration.

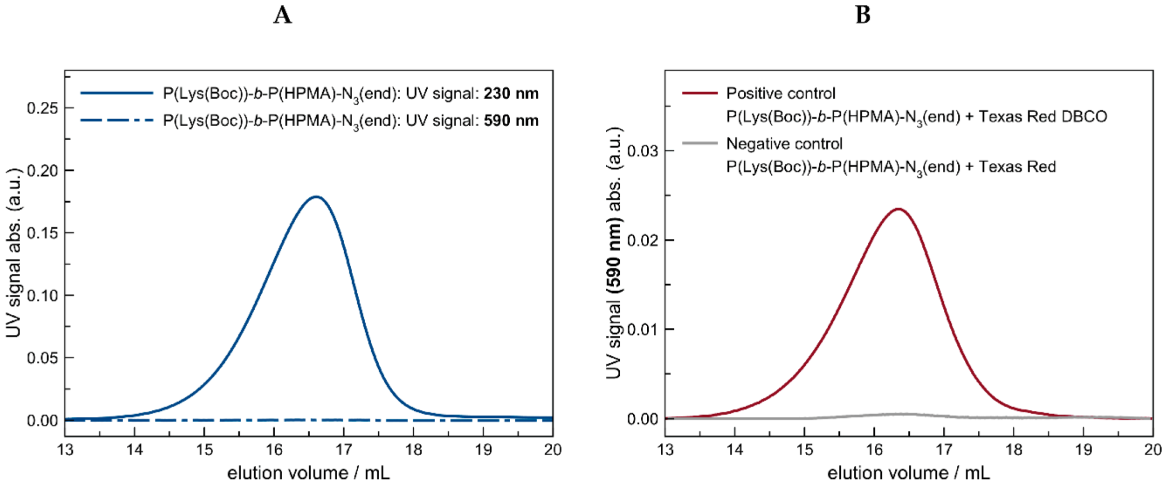

Figure S2. GPC in HFIP **A)** P(Lys(Boc))-*b*-P(HPMA)-N_3(end) at 230 nm (blue solid line) and 590 nm (blue dotted line). **B)** Azide detection reaction: SPAAC with Texas Red DBCO; positive control at 590 nm (red solid line), negative control at 590 nm (grey solid line).

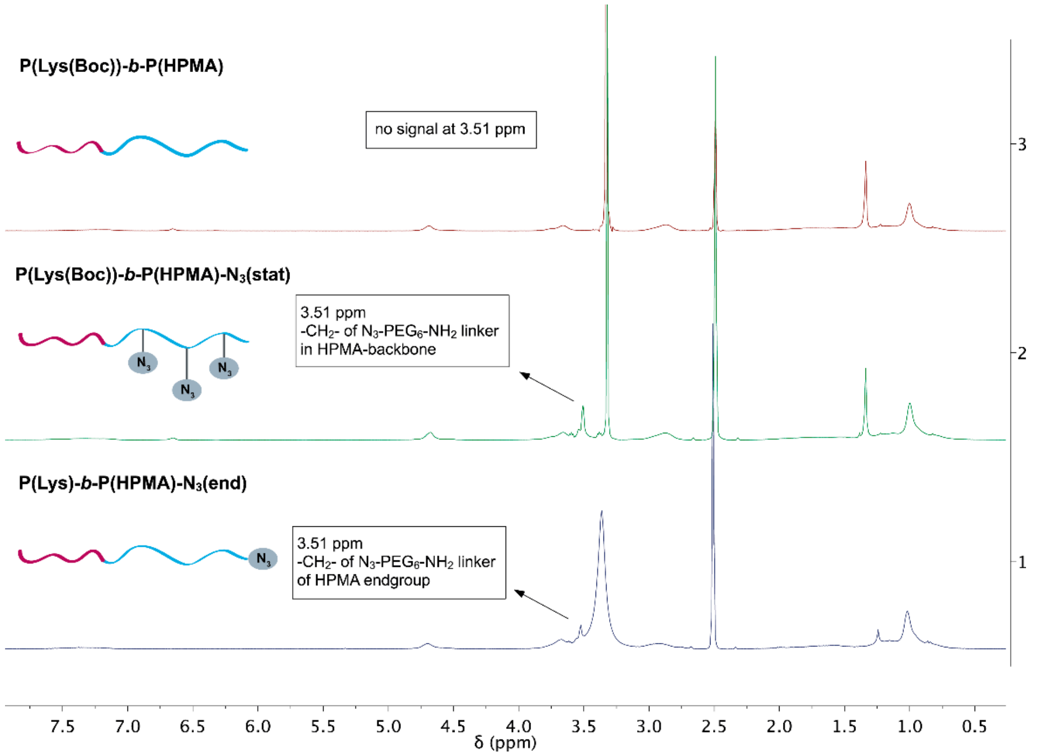

Figure S3. ^{1}H NMR spectra of P(Lys(Boc))-*b*-P(HPMA) (top), P(Lys(Boc))-*b*-P(HPMA)-N_3(stat) (middle) and P(Lys)-*b*-P(HPMA)-N_3(end) (bottom) in DMSO-d_6.

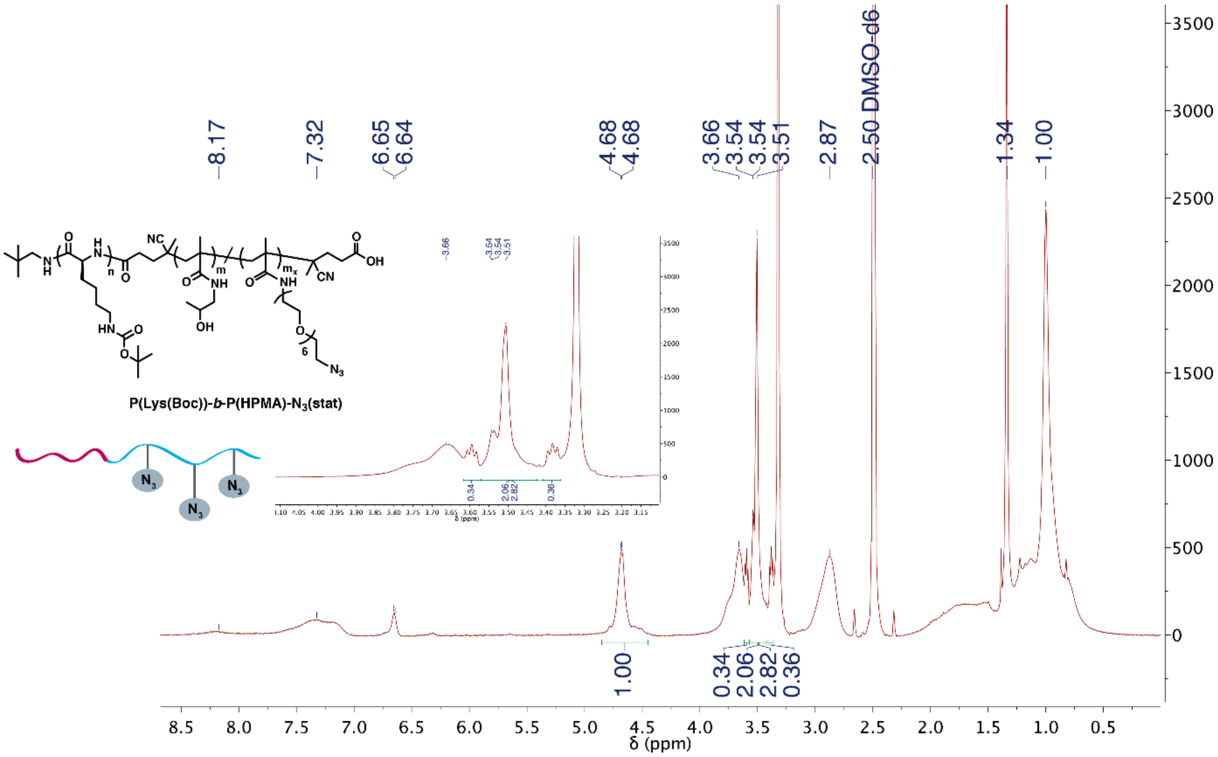

Figure S4. ^{1}H NMR spectra of P(Lys(Boc))-*b*-P(HPMA)-N_3(stat) in DMSO-d_6.

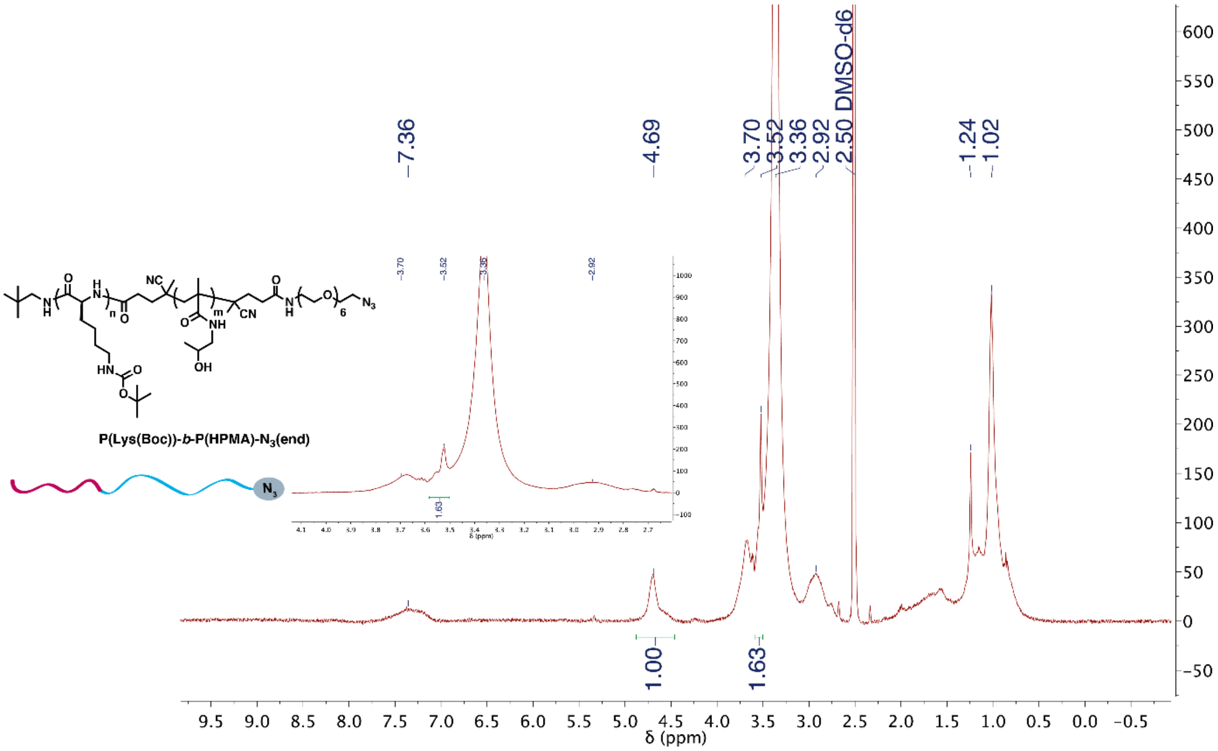

Figure S5. ^{1}H NMR spectra of P(Lys(Boc))-*b*-P(HPMA)-N_3(end) in DMSO-d_6.

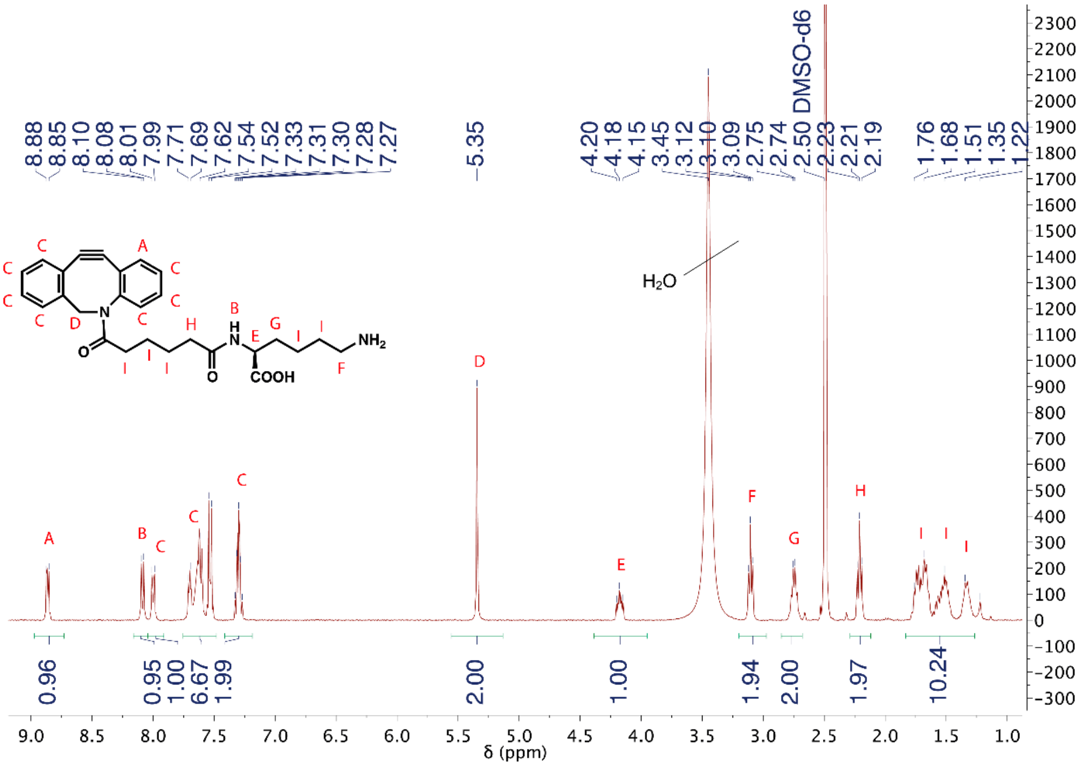

Figure S6. ^{1}H NMR spectra of DBCO-Lys in DMSO-d_6.

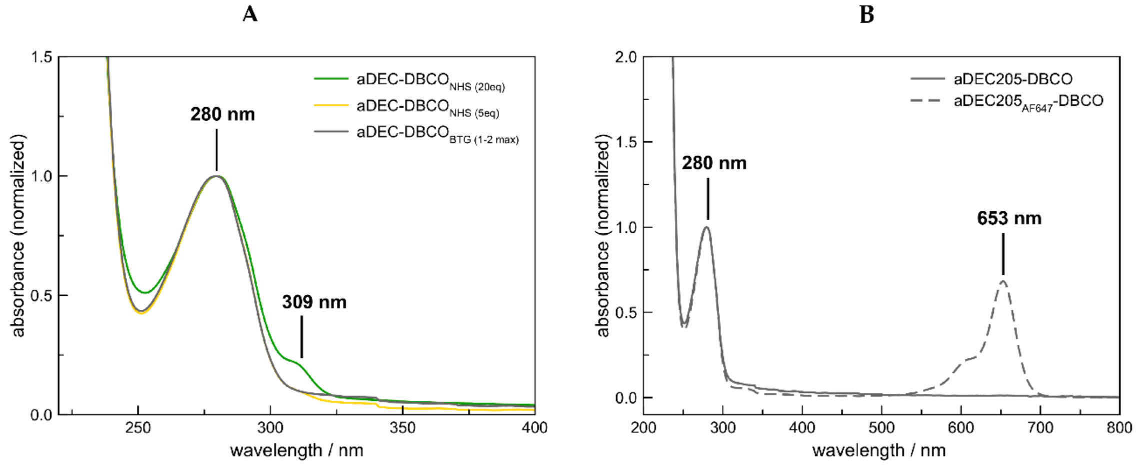

Figure S7. UV/Vis spectra **A)** Different aDEC205-DBCO species. **B)** aDEC205-DBCO (solid line) and aDEC205$_{AF647}$-DBCO (dotted line).

Sodium Dodecyl Sulfate Polyacrylamide Gel Electrophoresis: SDS PAGE

In a typical experiment, 1 µg of the respective protein sample (native aDEC205, PNGase F, BTG or aDEC205-DBCO) was filled up to a total volume of 7.5 µL with PBS (pH 7.4) and additional 2.5 µL of LDS sample buffer were added. The final volume of 10 µL for each protein sample was transferred into the SDS Gel pocket (NuPAGE™ 4-12% Bis Tris Protein Gels). For size determination 10 µL of Novex® Sharp Pre-Stained Protein Standard were loaded into each gel. Electrophoresis proceeded at 120 V for 30 min. Subsequent protein staining was performed using Coomassie Brilliant Blue R-250 for 2 h while gently shaking.

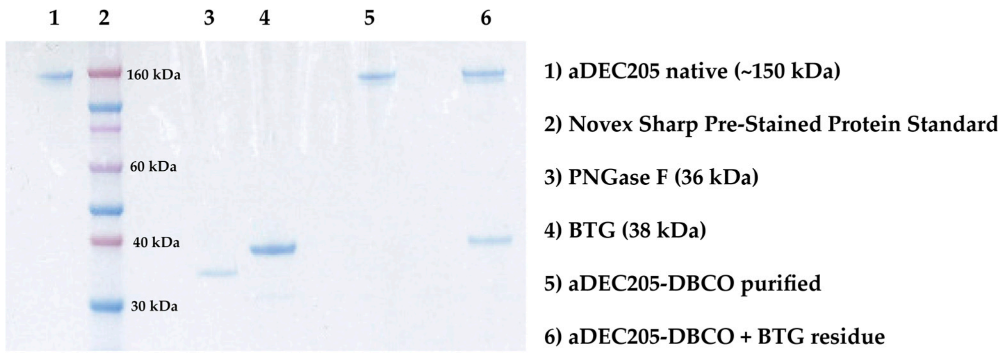

Figure S8. SDS PAGE of enzymatically DBCO-modified DEC205 antibody.

Dynamic Light Scattering: DLS

For multi-angle dynamic light scattering (DLS) cylindrical quartz cuvettes (Hellma, Mühlheim, Germany) were cleaned by dust-free distilled acetone and transferred to a dust free flow box. Sample solutions were filtered into the cuvettes through Pall GHP filters, 0.2 µm pore size. DLS measurements were performed by the following instrument at 20 °C. The apparatus consists of a Uniphase He/Ne Laser (25 mW output power at λ=632.8 nm) and an ALV-CGS 8F SLS/DLS 5022F goniometer with eight simultaneously working ALV 7004 correlators and eight QEAPD avalanche photodiode detectors. The correlation functions of the particles were fitted using a sum of two exponentials. The z-average diffusion coefficient D_z was calculated by extrapolating D_{app} for q=0. By formal application of Stokes law, the inverse z-average hydrodynamic radius is $R_h = \langle R_h^{-1} \rangle_z^{-1}$.

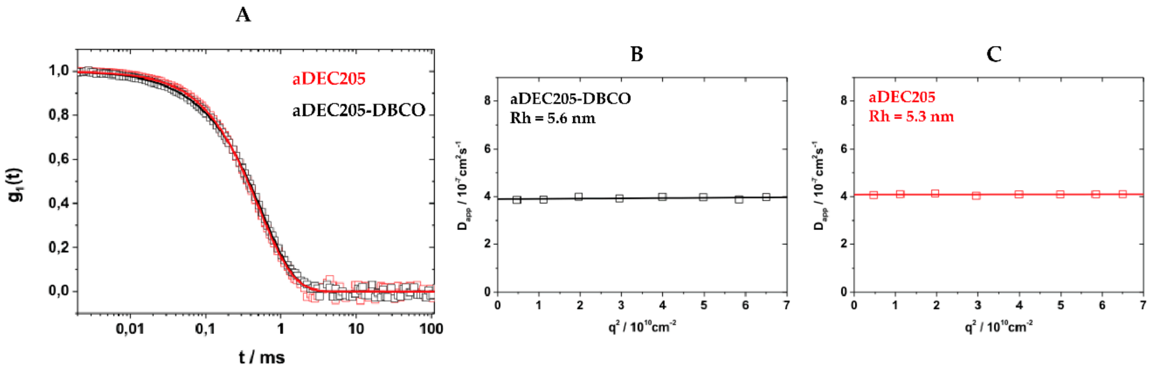

Figure S9. DLS: **A)** Autocorrelation function of aDEC205 (red line) and aDEC205-DBCO (black line). **B)** Multi angle DLS: aDEC205-DBCO (R_h=5.6 nm). **C)** Multi angle DLS: aDEC205 (Rh=5.3 nm).

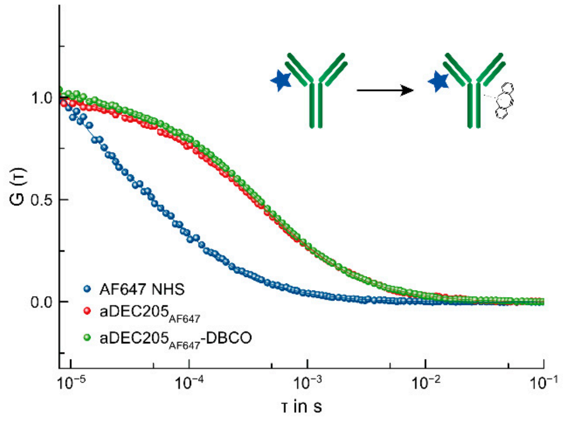

Figure S10. FCS: Comparison of $aDEC205_{AF647}$ and $aDEC205_{AF647}$-DBCO**:** blue line: AF647 NHS dye: R_h=0.76 nm; red line: $aDEC205_{AF647}$: R_h=6.0 nm; green line: $aDEC205_{AF647}$-DBCO: R_h=6.0 nm.

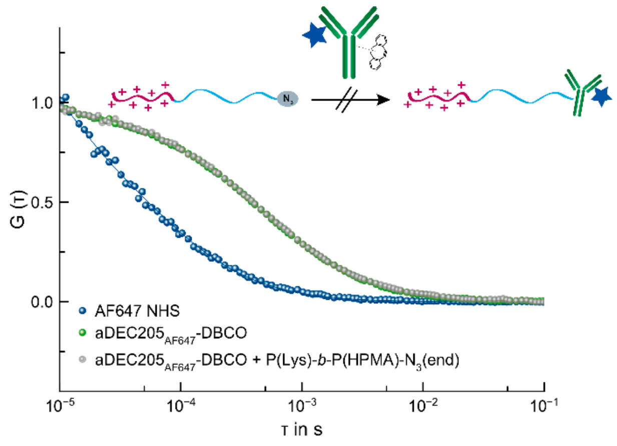

Figure S11. FCS: SPAAC of $aDEC205_{AF647}$-DBCO and P(Lys)-*b*-P(HPMA)-N_3(end): blue line: AF647 NHS dye: R_h=0.76 nm; green line: $aDEC205_{AF647}$-DBCO: R_h=6.0 nm; grey line: failed SPAAC of $aDEC205_{AF647}$-DBCO and P(Lys)-*b*-P(HPMA)-N_3(stat): R_h=7.5 nm.

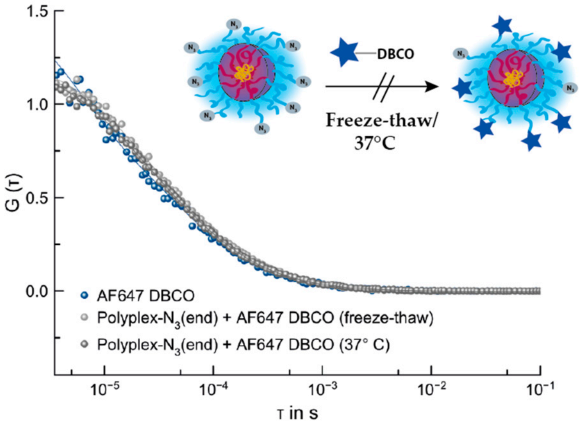

Figure S12. FCS: SPAAC of polyplex by P(Lys)-*b*-P(HPMA)-N_3(end) and AF647 DBCO: blue line: AF647 DBCO: R_h=0.75 nm; light grey line: failed SPAAC of polyplex: P(Lys)-*b*-P(HPMA)-N_3(end) and AF647-DBCO by freeze-thaw cycles; dark grey line: failed SPAAC of polyplex: P(Lys)-*b*-P(HPMA)-N_3(end) and AF647-DBCO at 37 C.

References

1. Heller, P.; Weber, B.; Birke, A.; Barz, M. Synthesis and sequential deprotection of triblock copolypept(o)ides using orthogonal protective group chemistry. *Macromol. Rapid Commun.* 2015, *36*, 38–44, doi:10.1002/marc.201400466.

2. Tappertzhofen, K.; Weiser, F.; Montermann, E.; Reske-Kunz, A.; Bros, M.; Zentel, R. Poly- l -Lysine-Poly[HPMA] Block Copolymers Obtained by RAFT Polymerization as Polyplex-Transfection Reagents with Minimal Toxicity. *Macromol. Biosci.* 2015, *15*, 1159–1173, doi:10.1002/mabi.201500022.

3. Jochum, F. D.; Borg, L. Zur; Roth, P. J.; Theato, P. *Macromolecules*. 2009, pp. 7854–7862.

4. Eberhardt, M.; Mruk, R.; Zentel, R.; Théato, P. Synthesis of pentafluorophenyl(meth)acrylate polymers: New precursor polymers for the synthesis of multifunctional materials. *Eur. Polym. J.* 2005, *41*, 1569–1575, doi:10.1016/j.eurpolymj.2005.01.025.
